# Spaceborne snapshot compressive hyperspectral imaging

**DOI:** 10.1038/s41377-026-02296-4

**Published:** 2026-05-18

**Authors:** Zhenming Yu, Liming Cheng, Jingyue Ma, Jiayu Di, Liang Lin, Ning Zhan, Tongshuo Zhang, Xing Zhong, Xiaojun He, Shanbo Chen, Xiaoxue Gong, Xu Cao, Huibin Zhang, Bingli Guo, Yongli Zhao, Shanguo Huang, Kun Xu

**Affiliations:** 1 State Key Laboratory of Information Photonics and Optical Communications, Beijing University of Posts and Telecommunications, Beijing, China; 2 Xiong’an Aerospace Information Research Institute, Xiong’an, China; 3 Chang Guang Satellite Technology Co., Ltd., Changchun, China

**Keywords:** Imaging and sensing, Optical spectroscopy

## Abstract

Hyperspectral remote sensing images provide rich spatial and spectral information about the Earth’s surface, making them an essential tool for Earth observation. However, existing spaceborne hyperspectral payloads experience slow acquisition speeds and generate large data volumes, posing significant challenges for real-time applications. Moreover, the complex optical design and relatively high cost of traditional hyperspectral payloads hinder their broad-scale in-orbit deployment. In this work, we have proposed and completed the world’s first computational imaging-enabled compact spaceborne snapshot compressive hyperspectral payload, named *BUPT-spectra01*, which was successfully launched on November 11, 2024, at the Jiuquan Satellite Launch Center in China. We design a reflective coding structure, which enables *BUPT-spectra01* to achieve high compactness (182 mm × 214 mm × 94 mm, 1.535 kg) and low cost. The payload operates in a sun-synchronous orbit at an altitude of 520 km, with a ground imaging swath width of 51 km by 64 km. Through a single exposure (1 ms), the payload enables 47-band hyperspectral imaging with a spectral resolution of 6.5 nm, achieving 47-times data compression simultaneously. To achieve high-accuracy hyperspectral information reconstruction, we design a novel spatial-spectral inference neural network (SSI-Net). Moreover, *BUPT-spectra01* can image at a rate of 30 frames per second, which allows video-level hyperspectral observation. In-orbit experiments demonstrate that *BUPT-spectra01* achieves accurate classification of ground cover based on hyperspectral features, showing promise in hyperspectral observation applications such as disaster management, environment monitoring, and resource exploration. This breakthrough significantly advances the application of computational imaging in aerospace observation, contributing to the progress of future satellite internet.

## Abstract (web summary)

We propose and complete the world’s first computational imaging-enabled compact spaceborne snapshot compressive hyperspectral payload, named BUPT-spectra01, which could achieve 30-fps, 47-band video-rate hyperspectral remote sensing in-orbit.

## Graphical abstract

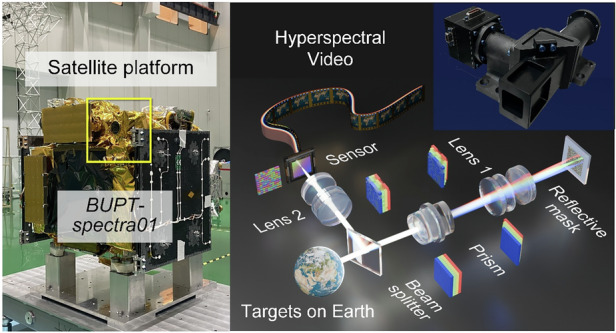

## Introduction

As a vital tool in Earth observation, hyperspectral remote sensing (HRS) acquires dozens or even hundreds of continuous-wavelength narrow-band images of the Earth’s surface. Unlike multispectral remote sensing, HRS provides detailed spectrum fingerprints of materials, enabling precise identification and discrimination of ground cover. Due to these advantages, HRS can be extensively applied across various fields, such as disaster management^1,2^, environment monitoring^3–5^, and resource exploration^6–8^.

Existing spaceborne hyperspectral imagers are mainly categorized into dispersion-based and filter-based types. The dispersion-based pushbroom spaceborne hyperspectral imagers (such as PRISMA^9^, HISUI^10^, and EnMAP^11^) first separate the incoming light into different wavelengths using prisms or gratings. Then they capture the spectrum of a line area on the Earth’s surface in one exposure and scan the hyperspectral data along the track of satellites. However, in dispersion-based pushbroom hyperspectral imagers, the optical flux per spectral channel significantly decreases after dispersion, particularly with increasing spectral bands. To achieve high spatial resolution, a larger aperture is typically required, leading to complex optical designs and relatively high cost^12–14^. The spectral filter-based spaceborne hyperspectral imagers (such as HySI^15^ and HyperScout^16^) utilize spectral filters to separate the information of different wavelengths. A typical technique involves adopting linear variable filters in front of the detector arrays to filter spectral information along the gradient direction, which reduces both the volume and cost of hyperspectral imagers^17,18^. However, in satellite-to-ground scenarios with relative motion, the filter-based technology suffers from temporal delays when imaging the same target across different spectral bands. As a result, inter-spectral registration of fast-moving targets (e.g., planes) becomes challenging at high spatial resolutions^19^. Besides, both techniques face the following common dilemmas. First, due to the huge volume of hyperspectral data, satellite storage and data downlink face considerable challenges^20,21^. Second, as the two types of techniques are constrained by the time-consuming imaging process, they are unable to make dynamic observations^22,23^. As global hyperspectral observation progresses toward big data and high dynamics^24–26^, new advancements are needed to overcome these limitations.

Currently, snapshot compressive hyperspectral imaging (SCHI) technologies have emerged as a promising approach for enhancing hyperspectral imaging capabilities^27–39^. By utilizing compressive sensing theory^40,41^, SCHI encodes multidimensional hyperspectral information into single-shot two-dimensional measurements and reconstructs the hyperspectral data with advanced algorithms^42–44^. However, the SCHI technology has not been applied to spaceborne hyperspectral imaging due to several formidable challenges. Firstly, the ultra-long imaging distance imposes strict demands on the spectral imaging accuracy of the SCHI system. To achieve high ground resolution, a spaceborne observation system requires a long focal length. Under long focal length, tiny deviations in the relative position of the dispersive and coding elements can cause severe changes in the coding matrix, degrading the encoding quality. In addition, the spectral features are weak under ultra-long imaging distances, posing further challenges for accurate spectral reconstruction. Secondly, the harsh space environment exacerbates difficulties for its in-orbit deployment. Drastic temperature fluctuations and vacuum conditions can induce deformations in optical components, leading to degradation of imaging performance. Additionally, vibrations during rocket launch and micro-vibrations from in-orbit attitude adjustments can result in misalignment of system components, particularly the optical encoding elements, imposing exceptionally stringent demands on the structural stability of the SCHI systems. Lastly, the resource constraints of satellites place rigorous limitations on payload mass, volume, and power consumption, necessitating a highly optimized system design.

In this work, we have proposed and completed the world’s first computational imaging-enabled compact spaceborne snapshot compressive hyperspectral payload, named *BUPT-spectra01*, which was successfully launched at the Jiuquan Satellite Launch Center in China (Supplementary Information Section 1). We design a reflective hyperspectral encoding structure, which reduces the use of optical components in *BUPT-spectra01* through optical path multiplexing while maintaining high imaging quality. This allows *BUPT-spectra01* to realize high compactness (182 mm × 214 mm × 94 mm, 1.535 kg) at a low cost, significantly facilitating its deployment on satellite platforms. Operating in a sun-synchronous orbit at an altitude of 520 km, *BUPT-spectra01* has an imaging swath width of 51 km by 64 km with a spatial resolution of 50 m. Through a single exposure (1 ms), *BUPT-spectra01* enables 47-band hyperspectral imaging with a spectral resolution of 6.5 nm in the range of 400–700 nm. Moreover, the single-shot method allows the acquisition of 47 spectral bands in a single measurement, achieving 47-fold data compression at the hardware level. Compared with traditional methods that require full hyperspectral data acquisition and software-based compression, this approach directly captures optically compressed data, saving both storage and computation resources on satellite platforms. Moreover, 30-fps hyperspectral video imaging can be achieved with *BUPT-spectra01*. Through the specialized custom designs, precise machining, and rigorous testing, we enable *BUPT-spectra01* to perform stable imaging in harsh space environments and at ultra-long distances. The key innovations introduced in the SCHI payload for the first time are as follows. In order to alleviate chromatic aberration and geometric distortion under in-orbit imaging conditions, we designed two customized lens groups with five and six lenses, respectively. An Amici prism was introduced to provide sufficient dispersion, ensuring effective separation of spectral information across 47 bands. To ensure efficient and reliable video-rate hyperspectral imaging, we adopted a low-noise, high-sensitivity sensor and designed internal circuitry capable of automatic self-configuration upon power-up. To suppress the interference of stray light on the weak spectral signal, a shading design was proposed at the light entrance, and high-absorption matting paint was applied to key components. To address focal shifts caused by lens expansion in a vacuum environment, we proposed a pre-compensation strategy and validated its effectiveness through vacuum chamber tests. Considering the risk of coding component displacement caused by mechanical vibrations during rocket transportation and launch, we proposed an integrated structural design using high-strength materials and anti-loosening mechanisms, which was verified through ground-based vibration tests. Furthermore, we performed radiometric calibration and modulation transfer function (MTF) tests to ensure accurate in-orbit hyperspectral imaging. Inspired by recent spectral reconstruction approaches^45–48^, we designed a spatial-spectral inference neural network (SSI-Net, Supplementary Information Section 2) for high-accuracy hyperspectral information recovery, which provides stronger spectral–spatial correlation modeling and multi-scale feature extraction capabilities. An on-ground test demonstrates that *BUPT-spectra01* achieves high-accuracy hyperspectral imaging. In-orbit imaging results further validate that the payload can achieve precise ground cover classification and video-level hyperspectral imaging, verifying its capability for high spatial–spectral–temporal resolution imaging. We anticipate this work will advance the application of computational imaging in aerospace observation and support various real-time Earth observation tasks, contributing to the development of future satellite internet.

## Results

### Principle and design

**Fig. 1:**
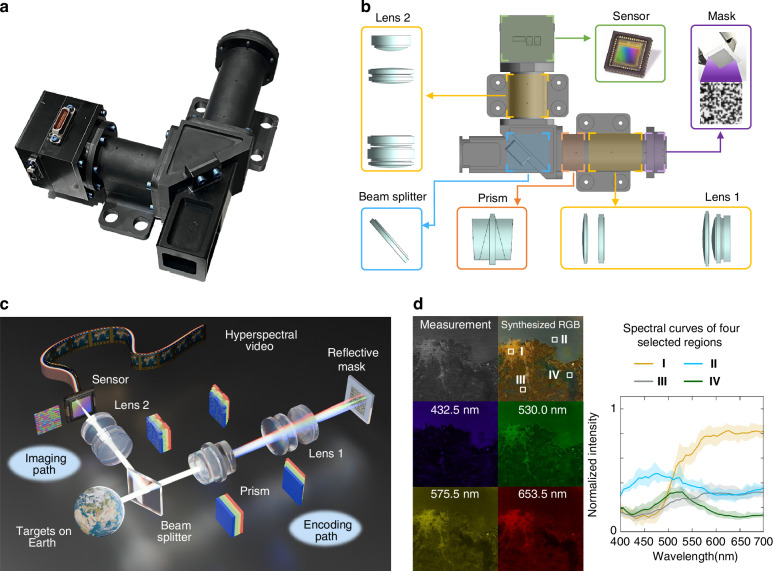
Working principle and the payload design. **a** A photograph of *BUPT-spectra01*. **b** The architecture of *BUPT-spectra01*. **c** The imaging principle of *BUPT-spectra01*. **d** The in-orbit hyperspectral imaging results of Montevideo, Uruguay, (34.606° S, 56.216° W). We plot the spectral curves of four spatial locations (I, II, III, IV). The solid curve represents the average spectrum of the regions, and the shaded area represents the spectral fluctuations within the regions. For each region, the upper bound is defined as the maximum spectral intensity among all pixels, while the lower bound is similarly determined by the minimum spectral intensity

Figure 1a shows a photograph of *BUPT-spectra01*. The payload architecture is illustrated in Fig. 1b. We design a reflective encoding structure for *BUPT-spectra01* (Fig. 1c). The hyperspectral information first enters the encoding path and is dispersed by the prism, resulting in spatial shifts between different spectral bands. On the mask plane, the hyperspectral data is modulated and reflected by the reflective mask. Then the data returns back to the encoding path and passes through the prism again. Compared to single-dispersion SCHI systems^48^, the second dispersion eliminates the spectral shifts to reduce spatial distortion caused by the prism’s nonlinear dispersion, thereby enhancing spatial imaging quality. Furthermore, compared with the dual-disperser architecture in ref. ^49^, *BUPT-spectra01* achieves a significant reduction in system volume through optical path multiplexing, which is particularly suitable for deployment on satellites with limited space. Finally, the hyperspectral information is directed into the imaging path by the beam splitter and captured by a sensor as a two-dimensional optical measurement. The mathematical model is described in Methods. Notably, *BUPT-spectra01* collects light from the Earth, which is approximately at an infinite distance. Therefore, we reduce the usage of an objective lens in front of the beam splitter, further improving the compactness of *BUPT-spectra01*. To reduce the interference from out-of-band information, all the glass components are coated with an anti-reflection film for the 400–700 nm wavelength range. We adopt an Amici prism to ensure sufficient spectral dispersion so that the sensing matrices of any two adjacent spectral bands do not overlap, resulting in smaller spectral reconstruction errors. Additionally, we adopt five lenses for lens 1 and six lenses for lens 2 to further reduce imaging aberrations and distortions. The image sensor (EV76C661) consists of 1280 × 1024 pixels and has a pixel size of 5.3 μm. The mask is a binary random pattern with 1500 × 1500 pixels, fabricated on a glass substrate. We adopt a binary coded aperture because it performs simple intensity modulation, which simplifies system calibration and enhances system reliability. Moreover, this design is less sensitive to manufacturing errors and system noise, which reduces risks during on-orbit deployment and is particularly well-suited for aerospace observation applications. Since containing more pixels than the sensor, the mask provides full coverage of the sensor plane, thereby eliminating the requirement for precise alignment during assembly. Each unit is a 10.6 μm square, covering a 2 × 2 region of sensor pixels. This design helps enhance hyperspectral imaging performance^50^. Despite the mismatch in pixel size between the mask and the sensor, no scaling operation is required, and the final imaging resolution is determined by the sensor. The silver- coated “1” units reflect the incident light, while the uncoated “0” units transmit the incident light. On the mask plane, the intensity of the hyperspectral image is modulated by the binary matrix, and the hyperspectral data is compressively sampled. During imaging, the mask is kept at rest. Moreover, a layer of silicon dioxide is added on the mask surface to ensure that the mask is not oxidized during ground testing and rocket transportation, thereby ensuring the quality of subsequent in-orbit coding. Figure 1d shows the hyperspectral imaging results of Montevideo, Uruguay, obtained at (34.606° S, 56.216° W). Fine spatial details can be clearly observed in the reconstructed hyperspectral images. Additionally, we plot the spectral curves of four spatial locations marked in the synthesized RGB image. Based on the distinct spectral intensity differences, different types of ground cover can be effectively distinguished. These results verify that BUPT-spectra01 can acquire rich spatial and spectral details. The 47-band images of Montevideo are provided in Supplementary Information Section 3.

### Image quality assessment

**Fig. 2:**
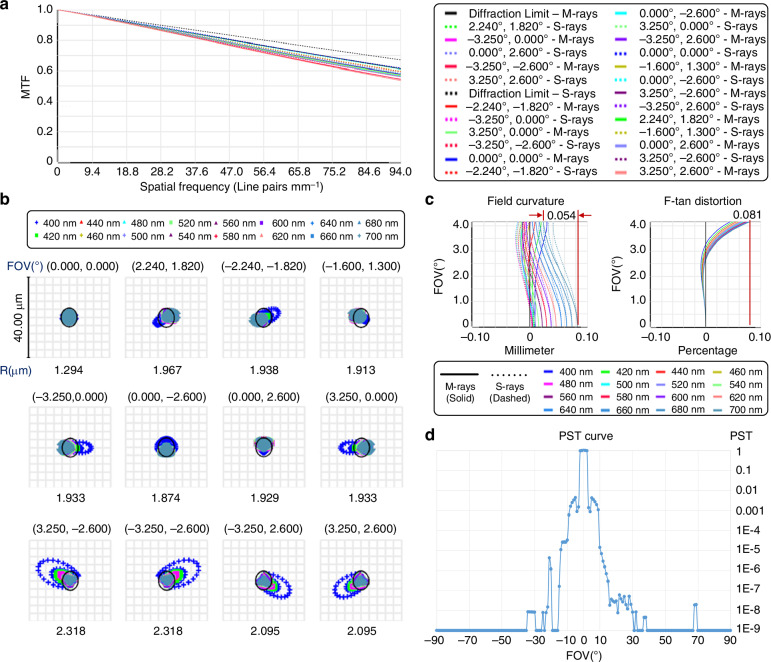
Image quality assessments. **a** The modulation transfer function (MTF) of meridional rays (M-rays) and sagittal rays (S-rays) at different fields of view (FOVs) and at the diffraction limit. **b** The spot diagrams of 16 wavelengths on the image plane at 12 FOVs. R is the average radius of speckles over 16 wavelengths. **c** The field curvature (left) and the F-tan distortion (right) at different FOVs ranging from 0° to 4.162°. **d** The point source transmission (PST) at different FOVs ranging from −90° to 90°

To validate the image quality of *BUPT-spectra01*, we conducted a series of assessments based on ZEMAX, as shown in Fig. 2. Figure 2a shows the MTF of meridional and sagittal rays at different fields of view (FOVs) and at the diffraction limit. When the Nyquist frequency of the detector is 94 $\mathrm{lp}\,{\mathrm{mm}}^{-1}$, the average MTF over all FOVs is 0.58, which indicates the excellent imaging resolution and sharpness of *BUPT-spectra01*. Furthermore, ground-based MTF tests were conducted after the payload was assembled (see Supplementary Information Section 4). Figure 2b shows the spot diagrams of 16 wavelengths on the image plane at 12 FOVs. Over all FOVs, the average speckle radius R of 16 wavelengths is less than one image sensor pixel (5.3 μm), demonstrating that the aberrations are effectively eliminated. Figure 2c illustrates that from 0° FOV to 4.162° FOV, the maximum field curvature and distortion are controlled to 0.054 mm and 0.081%, respectively. Finally, to eliminate the impact of stray light, we employ a shading design at the light entrance and spray high-absorption matting paint on the key components. For quantitative evaluation, we measure the point source transmittance (PST) at different FOVs, as shown in Fig. 2d. At near-field angles, the high PST is caused by reflections between the lenses and cannot be avoided. At other angles, the PST is generally controlled below ${10}^{-5}$, indicating that the stray light is effectively suppressed. These assessments demonstrate that *BUPT-spectra01* exhibits superior optical imaging quality and can effectively meet the demand for in-orbit hyperspectral observation tasks. Additionally, considering that “keystone” and “smile” distortions^51^ can significantly degrade imaging quality in conventional scanning hyperspectral imaging systems, we performed simulations to evaluate their impact in our system (Please see Supplementary Information Section 5).

### Performance and environmental testing on the ground

**Fig. 3:**
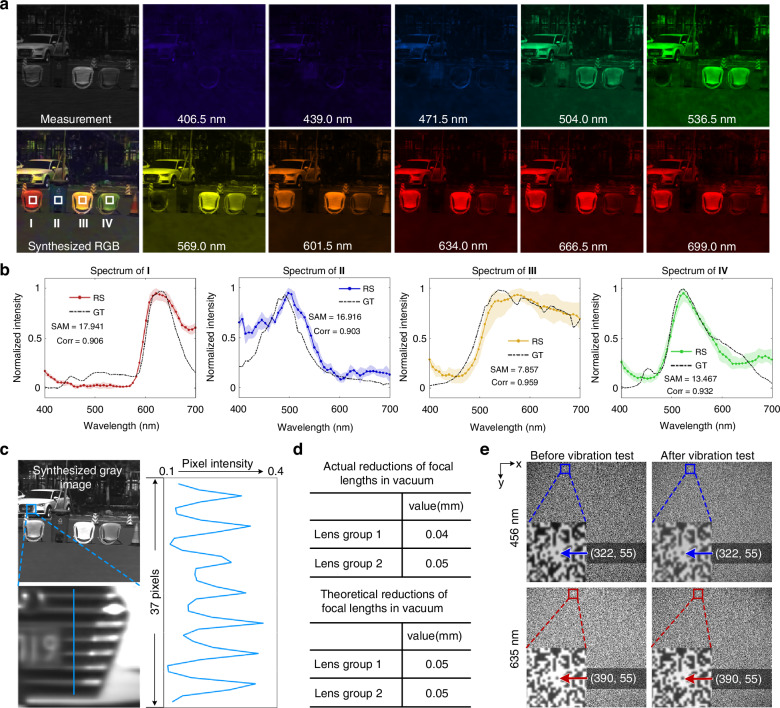
Performance and environmental testing of BUPT-spectra01 on the ground. **a** The hyperspectral imaging results on the ground. The synthesized RGB image and hyperspectral images of 10 bands are shown. **b** The reconstructed spectra (RS) and ground truth (GT) of four regions that are marked in the synthesized RGB. SAM: Spectral Angle Mapper. Corr: Pearson correlation coefficient. **c** The synthesized gray image. A region marked with a blue rectangle is zoomed for spatial evaluation. **d** The theoretical and actual reductions of focal lengths in a vacuum caused by the expansion of the lenses. **e** The transmission matrices of 456 nm and 635 nm before and after the vibration test. The images are captured using monochromatic lasers to incident the payload. The four subfigures provide the coordinates of a “0” unit located at the center of the zoomed region

To validate the reliability of *BUPT-spectra01* for in-orbit hyperspectral observation, we conducted performance and environmental testing on the ground (for more details, see Supplementary Information Section 6). Figure 3a shows the hyperspectral imaging results of an outdoor scenario. The targets were placed 50 meters away from the payload, where the light emitted by the targets can be approximated as light from an infinite distance. The results demonstrate that *BUPT-spectra01* can acquire rich spatial and spectral details. Figure 3b shows the reconstructed spectra (RS) and the ground truth (GT) of four regions, which are marked in the synthesized RGB image. The GT was acquired with a commercial spectrometer (FLAME-T-UV-VIS-ES, Ocean Insight). To quantitatively evaluate the spectral reconstruction accuracy, we calculate the Spectral Angle Mapper (SAM)^52^ and the Pearson correlation coefficient (Corr)^53^ between the RS and the GT of all the marked regions. The SAMs are less than 20, and the Corrs are more than 0.9, validating the high spectral accuracy of *BUPT-spectra01*. Figure 3c evaluates the spatial accuracy of the payload. In the zoomed region, we plot the intensity of pixels along the blue line. The pixel intensity distribution validates the high spatial accuracy of *BUPT-spectra01*. In Supplementary Information Section 7, we provide more spatial evaluation results. Figure 3d shows the reduction of focal lengths in the vacuum caused by the expansion of the lenses. In a vacuum chamber, we measured the actual reductions of focal lengths as 0.04 mm (lens 1) and 0.05 mm (lens 2). To achieve vacuum compensation, we adjusted the mask and the sensor to the vacuum-state positions of the focal planes. The measured reductions show slight deviations from the theoretical calculation, which validates the effectiveness of vacuum compensation. Figure 3e shows transmission matrices of 456 nm and 635 nm before and after the vibration test. The details of the vibration test are provided in Supplementary Information Section 8. After a comprehensive vibration test, the mask and sensor will not experience pixel-level relative displacement, demonstrating that *BUPT-spectra01* is able to withstand the vibrations encountered during rocket transportation and launch.

### Test results in orbit

**Fig. 4:**
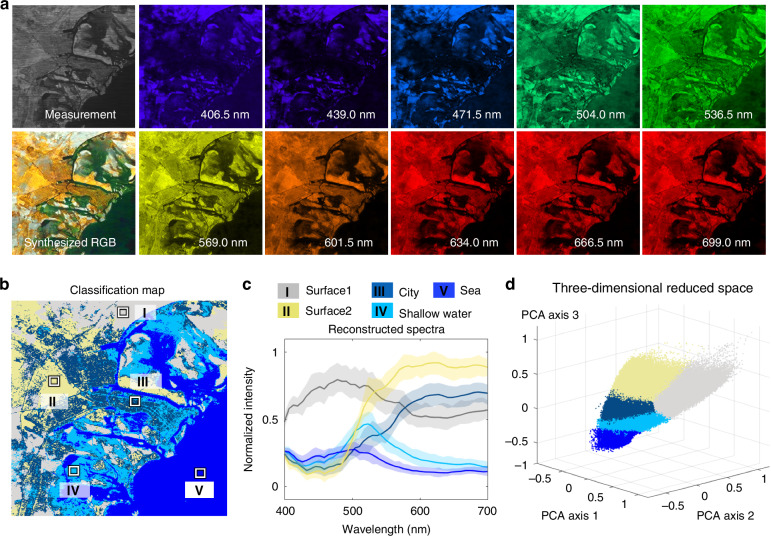
In-orbit hyperspectral imaging results of Abu Dhabi, UAE (24.418° N, 54.514° E). **a** The synthesized RGB image and the spectral images of 10 bands. **b** The classification map of ground cover using the K-means algorithm. **c** The reconstructed spectra of the five regions marked in the classification map. **d** The hyperspectral data distribution in three-dimensional reduced space using principal components analysis (PCA)

We conducted a series of in-orbit tests to validate the hyperspectral observation performance of *BUPT-spectra01*. Figure 4 shows the in-orbit hyperspectral imaging results obtained at Abu Dhabi, UAE (24.418° N, 54.514° E). Please see more in-orbit imaging results in Supplementary Information Section 9. In Fig. 4a, the synthesized RGB image and the spectral images provide rich spatial and spectral information, enabling precise recognition of ground cover. By applying the K-means algorithm^54^, we obtain a classification map that contains five types of ground cover, as shown in Fig. 4b. To evaluate the classification performance, we manually annotated a ground truth classification map based on high-resolution Google Maps imagery (Supplementary Information Section 10). By calculating the proportion of correctly classified pixels, we achieved a classification accuracy of 80.493%. According to the classification results, we plot the reconstructed spectra of five types of ground cover. As shown in Fig. 4c, we can observe that the five types of ground cover exhibit different spectral responses in the long-wavelength regime (600 nm–700 nm), validating that *BUPT-spectra01* can precisely acquire the spectral signatures of the Earth’s surface. For further validation, we adopt principal components analysis (PCA)^55^ along the spectral dimension to transform the 47-band hyperspectral data to a three-channel PCA image. In the three-dimensional reduced space (Fig. 4d), different types of ground cover are separated into different clusters. These results demonstrate the potential of *BUPT-spectra01* for urban planning and coastal zone management^56^.

**Fig. 5:**
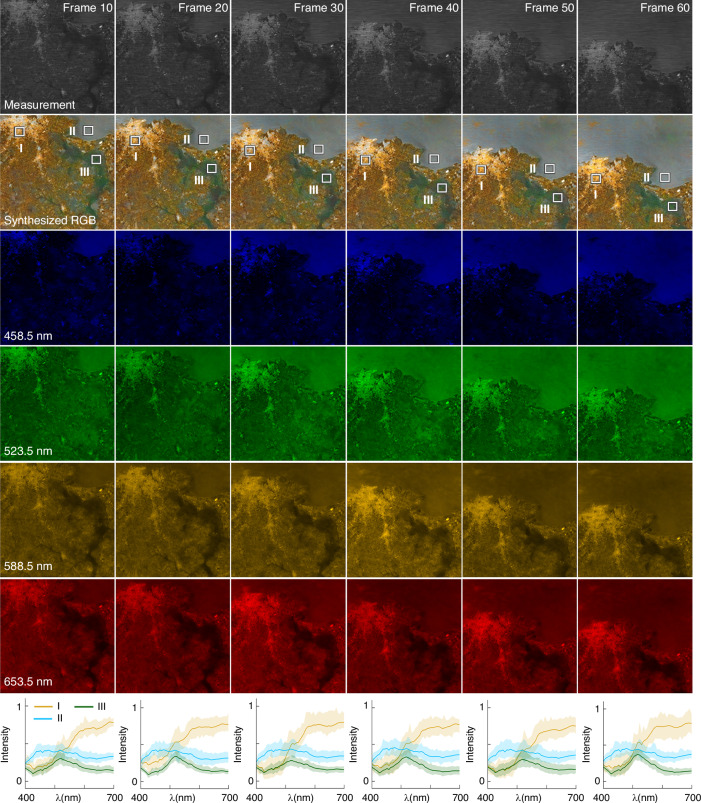
In-orbit hyperspectral video imaging results for Montevideo, Uruguay (34.606° S, 56.216° W). The synthesized RGB images and spectral images of four bands (458.5 nm, 523.5 nm, 588.5 nm, 653.5 nm) of six video frames are shown. The last row plots the reconstructed spectra of the three regions (I, II, III) marked in the synthesized RGB images. *λ*: wavelength

Figure 5 shows the 30-fps hyperspectral video imaging results of *BUPT-spectra01* that were obtained at Montevideo, Uruguay (34.606° S, 56.216° W). We choose six hyperspectral video frames for performance evaluation. The hyperspectral video is provided in Movie S1. At 458.5 nm wavelength, the coastline can be easily recognized due to the intensity difference between the sea and the land. At a long- wavelength regime, e.g., 653.5 nm, the city area (high intensity) and river valley (low intensity) can be easily distinguished. We plot the reconstructed spectra of three regions (I, II, III), which are marked in the synthesized RGB images, as shown in the last row. With the time variation, BUPT-spectra01 exhibits consistent spectral recovery. These results validate the stable spatial and spectral reconstruction performance of BUPT-spectra01 for in-orbit video-level hyperspectral imaging. By adjusting the optical design and adopting the staring imaging mode, the payload can achieve observations at higher spatial resolution (e.g., meter-level). At this finer scale, the snapshot imaging approach offers significant advantages for monitoring dynamic targets such as aircraft and ships. This method is particularly well-suited for future real-time remote sensing applications that require high-temporal-resolution hyperspectral imaging. More hyperspectral video results are provided in Movie S2.

## Discussion

We have proposed and completed the world’s first computational imaging-enabled compact spaceborne snapshot compressive hyperspectral payload, named *BUPT-spectra01*, which was successfully launched at the Jiuquan Satellite Launch Center in China. In-orbit experiments demonstrated that *BUPT-spectra01* can achieve 30-fps 47-band hyperspectral imaging. This work significantly advances the existing hyperspectral Earth observation systems toward high-temporal resolution, enabling dynamic hyperspectral monitoring for the Earth’s surface. Combined with real-time processing algorithms, *BUPT-spectra01* can provide more accurate assessments of dynamic events on the Earth’s surface, facilitating timely decision-making in disaster response and environmental monitoring scenarios. By further enhancing the spatial resolution to 5 m or better, this technology can acquire high spatial–spectral–temporal resolution information of ground objects, which can support the hyperspectral tracking of moving targets. Leveraging the optical domain data compression, *BUPT-spectra01* reduces the storage requirements and transmission bandwidth demands on satellite platforms. This capability allows hyperspectral imagers to be widely deployed on resource-constrained satellites, such as micro-nano satellites and CubeSats. For satellites with adequate resources, the compressed acquisition manner can support long-duration and large-scale observations, thereby enhancing the overall information throughput of space-based observation systems. We believe this work will enhance the global hyperspectral observation efficiency of existing space-based systems and contribute to the construction of a hyperspectral satellite constellation for future satellite internet.

## Materials and methods

### Mathematical model of *BUPT-spectra01*

The hyperspectral information from the Earth can be expressed as ${\rm{X}}({\rm{x}},\,{\rm{y}},\,{\rm{\lambda }})$, where $({\rm{x}},\,{\rm{y}})$ represents the spatial location and ${\rm{\lambda }}$ is the spectral location. After passing through the encoding path, the hyperspectral information is encoded to a two-dimensional measurement. This process is expressed as:

1 $$Y(x,y)={\int }_{{\lambda }_{\min }}^{{\lambda }_{\max }}X(x,y,\lambda )\cdot M(x,y-\alpha (\lambda -{\lambda }_{{\rm{c}}}))\cdot d\lambda +G(x,y)$$

Where $\alpha$ is the linear dispersion coefficient, and ${\lambda }_{{\rm{c}}}$ is the center wavelength. $M(x,y)$ denotes the pattern of the mask, and $G(x,y)$ is the noise term. $[{\lambda }_{min},{\lambda }_{max}]$ represents the working band of *BUPT-spectra01*. According to compressive sensing theory^36^, the acquisition process can be rewritten in a matrix form as:

2 $$B=HA+g$$

Where $B\in {{\boldsymbol{{\mathbb{R}}}}}^{(mn)\times 1}$ is the measurement, and $m,n$ are the pixelated spatial coordinates. $A\in {{\boldsymbol{{\mathbb{R}}}}}^{(kmn)\times 1}$ is the vectorized hyperspectral data, where $k$ denotes the number of discrete spectral bands. $H\in {{\boldsymbol{{\mathbb{R}}}}}^{(mn)\times (kmn)}$ is the sensing matrix that can be obtained from the pattern of the mask. $g\in {{\boldsymbol{{\mathbb{R}}}}}^{(mn)\times 1}$ is the discretized noise matrix. By applying computational reconstruction algorithms, the hyperspectral information $A$ can be recovered from the measurement $B$.

## Supplementary information

Supplemental Information

Supplemental Video 1

Supplemental Video 2

## Data Availability

The data that support the findings of this study and custom codes are available from the corresponding author upon reasonable request.
