# Supplementary material for: Spaceborne snapshot compressive hyperspectral imaging
Source: Light Sci Appl. 2026 May 18;15:234. doi: 10.1038/s41377-026-02296-4 (PMC13181047; doi:10.1038/s41377-026-02296-4)
Supplement: Supplementary file 1 — Supplemental Information [file 41377_2026_2296_MOESM1_ESM.docx]

**Supplementary Information for Spaceborne snapshot compressive hyperspectral imaging**

**Author list:** Zhenming Yu^1,2,4,*^, Liming Cheng^1,4^, Jingyue Ma^1^, Jiayu Di^1^, Liang Lin^1^, Ning Zhan^1^, Tongshuo Zhang^1^, Xing Zhong^3^, Xiaojun He^3^, Shanbo Chen^3^, Xiaoxue Gong^3^, Xu Cao^3^, Huibin Zhang^1^, Bingli Guo^1^, Yongli Zhao^1^, Shanguo Huang^1^, Kun Xu^1,2,*^.

^*^Corresponding author: yuzhenming@bupt.edu.cn; xukun@bupt.edu.cn

**Affiliation:**

^1^State Key Laboratory of Information Photonics and Optical Communications, Beijing University of Posts and Telecommunications, Beijing, China

^2^Xiong’an Aerospace Information Research Institute, Xiong’an, China.

^3^Chang Guang Satellite Technology Co., Ltd., Changchun, China.

^4^These authors contributed equally: Zhenming Yu and Liming Cheng.

**1. Information on the satellite platform and rocket launch**

The *BUPT-spectra01* payload was carried on the Jilin-1 02A03 satellite platform, as illustrated in Fig. S1a. The satellite was launched aboard the Kinetica-1 Y5 rocket (Fig. S1b).


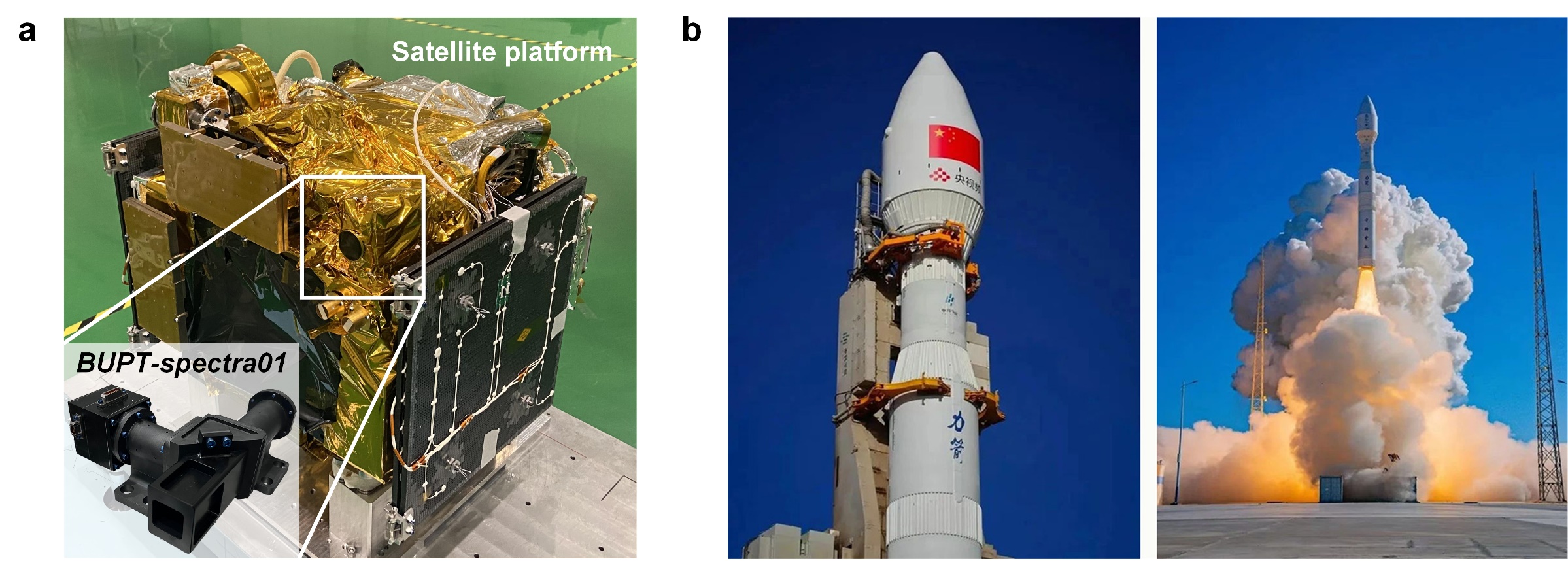


**Fig. S1. a,** Photograph of the satellite platform. **b,** Launch of the satellite aboard the Kinetica-1 Y5 rocket.

**2. Design of the SSI-Net**

In this section, we describe the network architecture of the SSI-Net (spatial-spectral inference neural network), As shown in Fig. S2a, the basic component of the SSI-Net is the SSI block, which focuses on capturing the dependencies between different spectral channels to enhance the hyperspectral reconstruction.

The first step is to generate the initialization of the network input. We illuminate the system with a monochromatic laser (635.0 nm) and capture the corresponding mask image on the sensor. This image is the sensing matrix of 635 nm wavelength. Due to uneven illumination, the mask image is brighter at the center and dimmer at the edges, resulting in lower calibration accuracy near the edges. Therefore, we crop the central

**
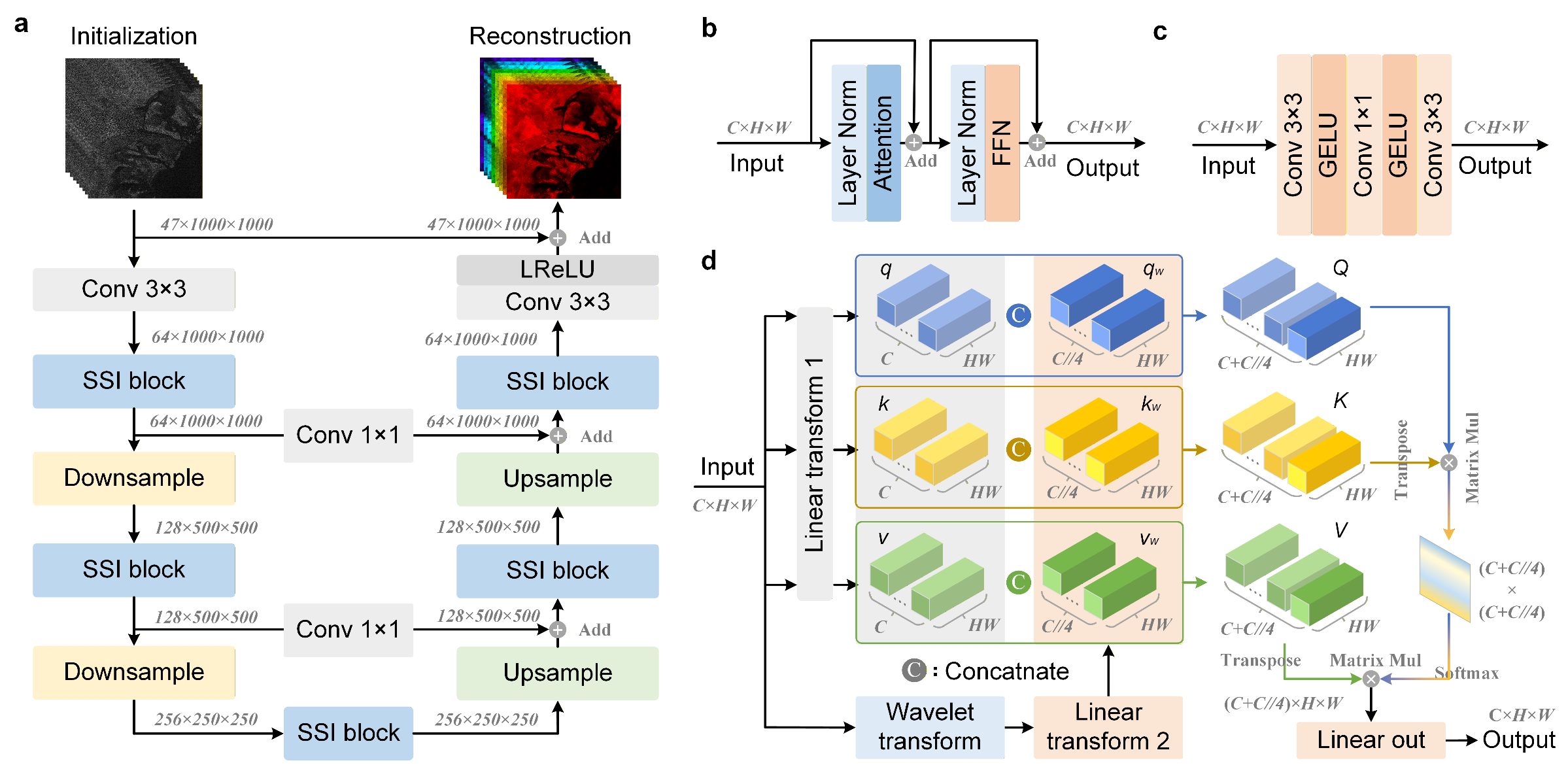
**

**Fig. S2 Details of the SSI-Net. a,** Structure of the SSI-Net. Conv: convolution. LReLU: leaky rectified linear unit. Add: addition. **b,** Structure of the SSI block. FFN: feed forward network. **c,** Structure of the FFN. GELU: Gaussian Error Linear Unit. **d,** Calculation of attention mechanism. Matrix Mul: matrix multiplication.

1000×1000 region for network reconstruction. Then, we shift the mask pattern along the dispersion direction to acquire all 47 sensing matrices, based on the dispersion differences between the spectral channels. Then, we duplicate the measurement 47 times and perform element-wise multiplication with the 47 transmission matrices to obtain the initialization data with a size of 47×1000×1000. Fig. S2b is the structure of the SSI block, which consists of two Layer Norm blocks [1,2], one FFN layer, and one Attention layer. The details of the FFN layer are shown in Fig. S2c. We adopt two 3×3 convolution blocks, one 1×1 convolution block, and two ReLU layers. The FFN layer allows the network to extract abundant hyperspectral features. Fig. S2d shows the structure of the Attention layer. For the input , we use the linear transform 1 layer to generate query (), key (), and value () matrices [1]. Meanwhile, we use wavelet transform to obtain rich frequency information of the input data. Then, the linear transform 2 layer maps the data to , , and . By concatenating the queries, keys, and values, we obtain the total query , total key , and total value . The attention map is calculated as:

$\mathrm{Atten}=\mathrm{softmax}\left(\text{αKQ}^{\mathrm{T}}\right)$ (1)

Where $\text{α}$ is a learnable parameter, and $\mathrm{softmax}$ is the activation function. Then, the output is calculated as:

$\text{Y}=\mathrm{reshape}\left(\mathrm{Linear} \mathrm{out}\left(\mathrm{Atten}\cdot\text{V}\right)\right)$ (2)

We use the CAVE hyperspectral dataset [3] to train the network and KAIST hyperspectral dataset [4] to validate the network performance. Mean square error (MSE) is adopted as the loss function:

$\mathrm{MSE}=\frac{1}{\text{N}}\left\|\hat{\text{x}}-\text{x}\right\|^{2}$ (3)

Where is the $\mathrm{L}_{2}$ norm, $\hat{\text{x}}$ is the reconstruction, $\text{x}$ is the ground truth, and *N* is the total number of pixels in the hyperspectral image $\text{x}$.

We compare the reconstruction performance of the proposed SSI-Net with several SOTA algorithms (Unet-3D [Manu. Ref. 31], TSA-Net [5], GAP-net [6], MST [7], HDNet [8]). The evaluation was conducted on 10 hyperspectral data in KAIST datasets. From the middle part of Table S1, we can observe that our SSI-Net achieves improvements in both PSNR and SSIM compared with the other SOTA methods. In addition, to provide a more comprehensive evaluation of the algorithm's performance, we assessed the total number of network parameters and the average reconstruction time per data sample. This assessment is conducted on a machine equipped with an Intel Xeon Gold 5218 CPU and two NVIDIA RTX 3090 GPUs. Compared to the SOTA methods, the proposed SSI-Net introduces a modest increase in model size and computation time. However, this overhead is acceptable and leads to clear performance gains.

**Table S1.** The middle part shows the PSNR evaluation (upper entry, unit: dB) and SSIM evaluation (lower entry) on 10 KAIST hyperspectral scenes. Avg: Average. The second to last column lists the total number of parameters for each algorithm. Para: Parameters. M: Million. The last column shows the average reconstruction time of each algorithm for the 10 hyperspectral data.


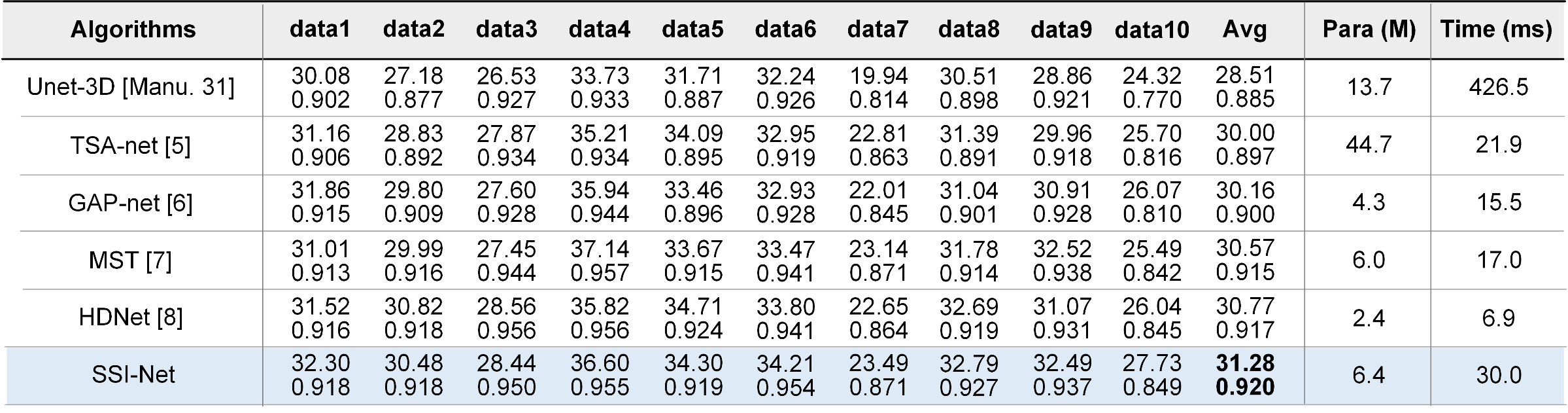


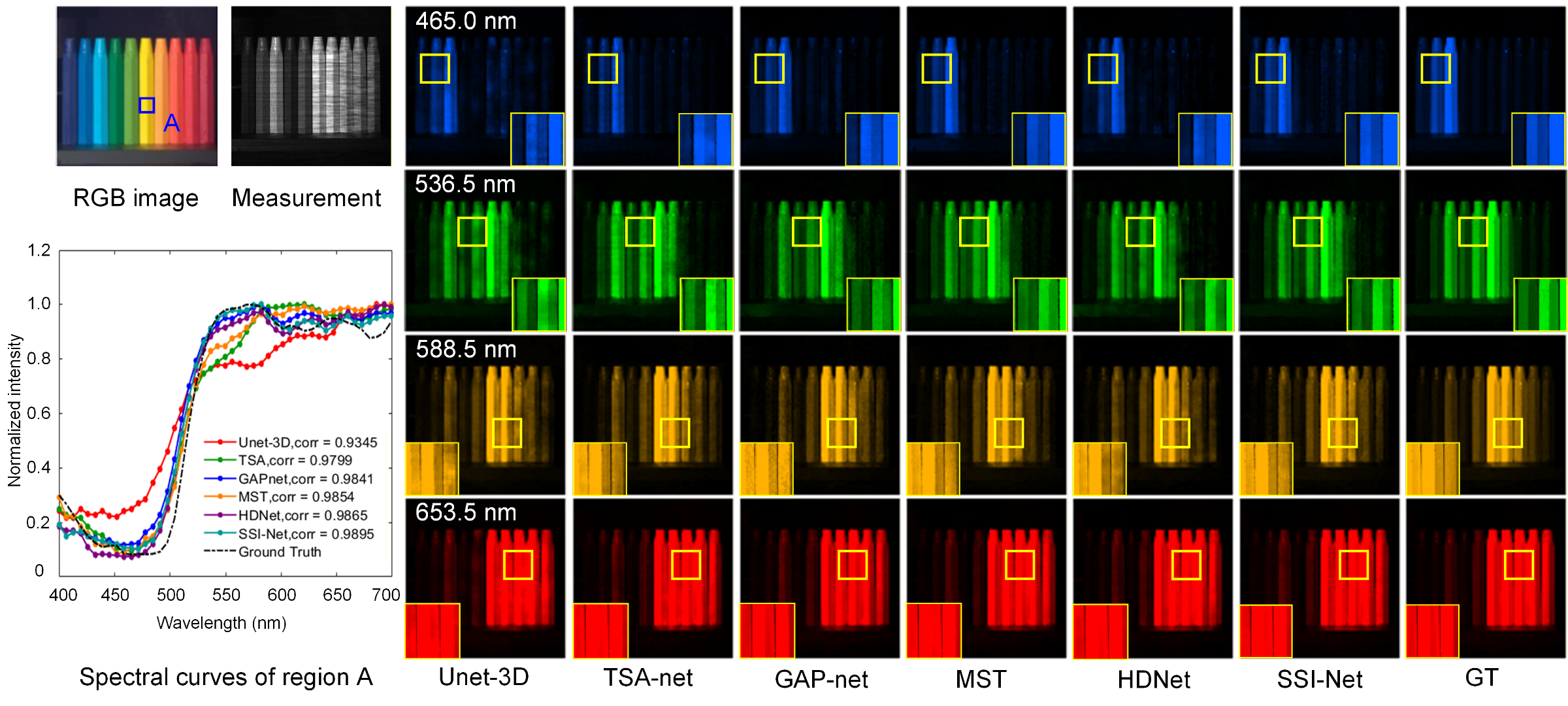


**Fig. S3. Reconstructed 4 band images of simulation hyperspectral scene**. 5 SOTA methods and out SSI-Net are included. The spectral curves (bottom left) of region A (marked in the RGB image) are plotted.

In Fig. S3, we show the reconstructed 4 band images of one scene for visual comparison. Our SSI-Net provide a slightly better visual performance. At the bottom left corner, we plot the spectral curves of region A (marked in the RGB image) achieved by these methods. The Pearson Correlation Coefficients (corr) are calculated for quantitative evaluation. We can observe that the SSI-Net achieves the highest corr of 0.9895. These results indicate that the proposed SSI-Net could achieve better spatial and spectral reconstruction.

**3. The 47-band hyperspectral images of Montevideo, Uruguay**

**
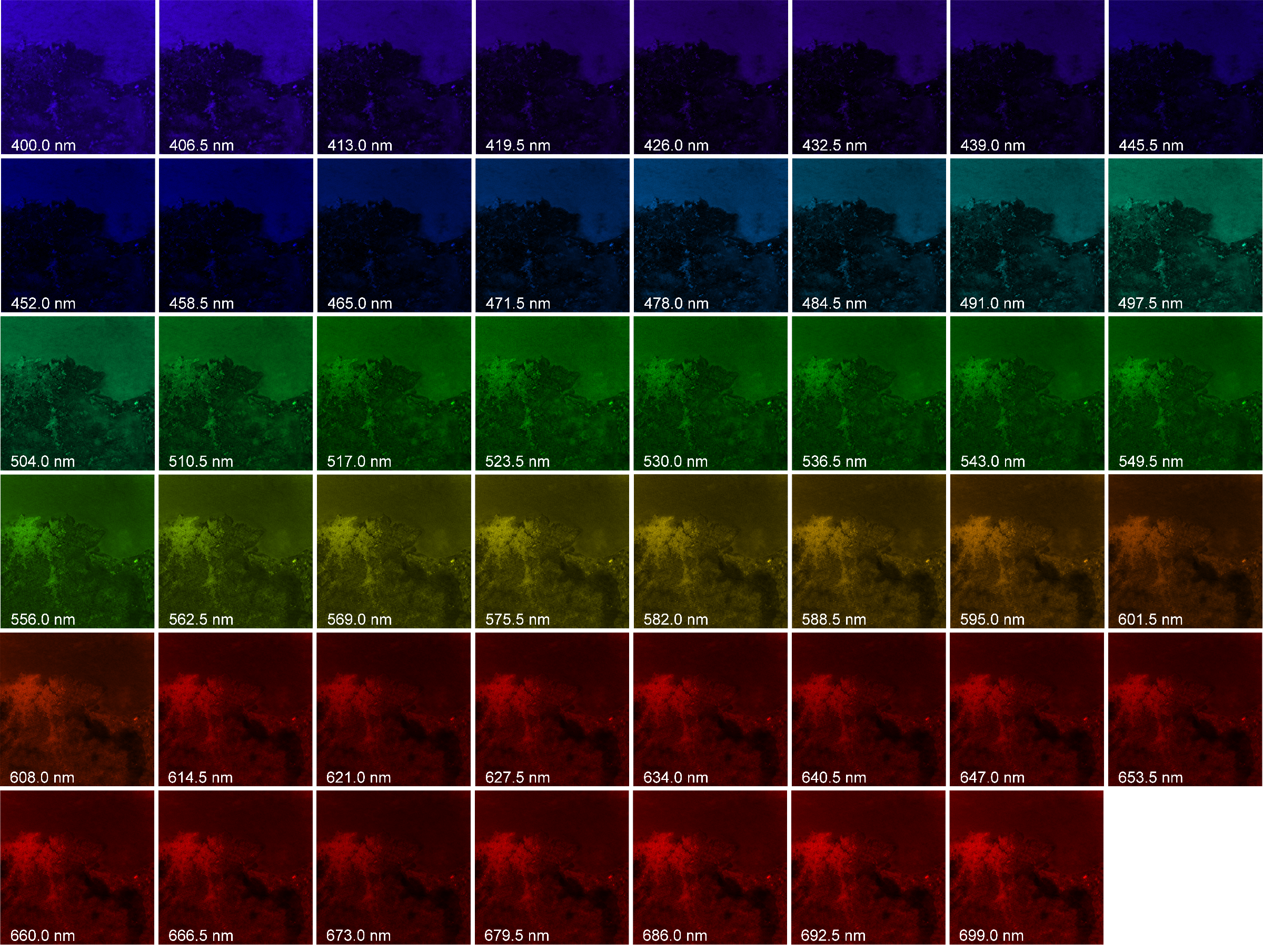
**

**Fig. S4.** The 47-band hyperspectral images of Montevideo, Uruguay (34.606° S, 56.216° W).

**4. Ground-based MTF tests**

This section presents the empirical MTF measurements of the payload prior to launch. We evaluated the MTF at different regions of the image plane. The nine subfigures shown in Fig. S5 correspond to the upper-left, upper-center, upper-right, middle-left, center, middle-right, lower-left, lower-center, and lower-right regions, respectively. The text above each subfigure shows the MTF value of a specified location. The results show that the MTF of the payload is approximately 0.3, which is mainly attributed to attenuation introduced during optical assembly and the attenuation of the detector. Although the MTF is lower than that obtained from the ZEMAX simulation, both the ground tests and on-orbit imaging results demonstrate that the payload can still achieve satisfactory hyperspectral imaging performance.


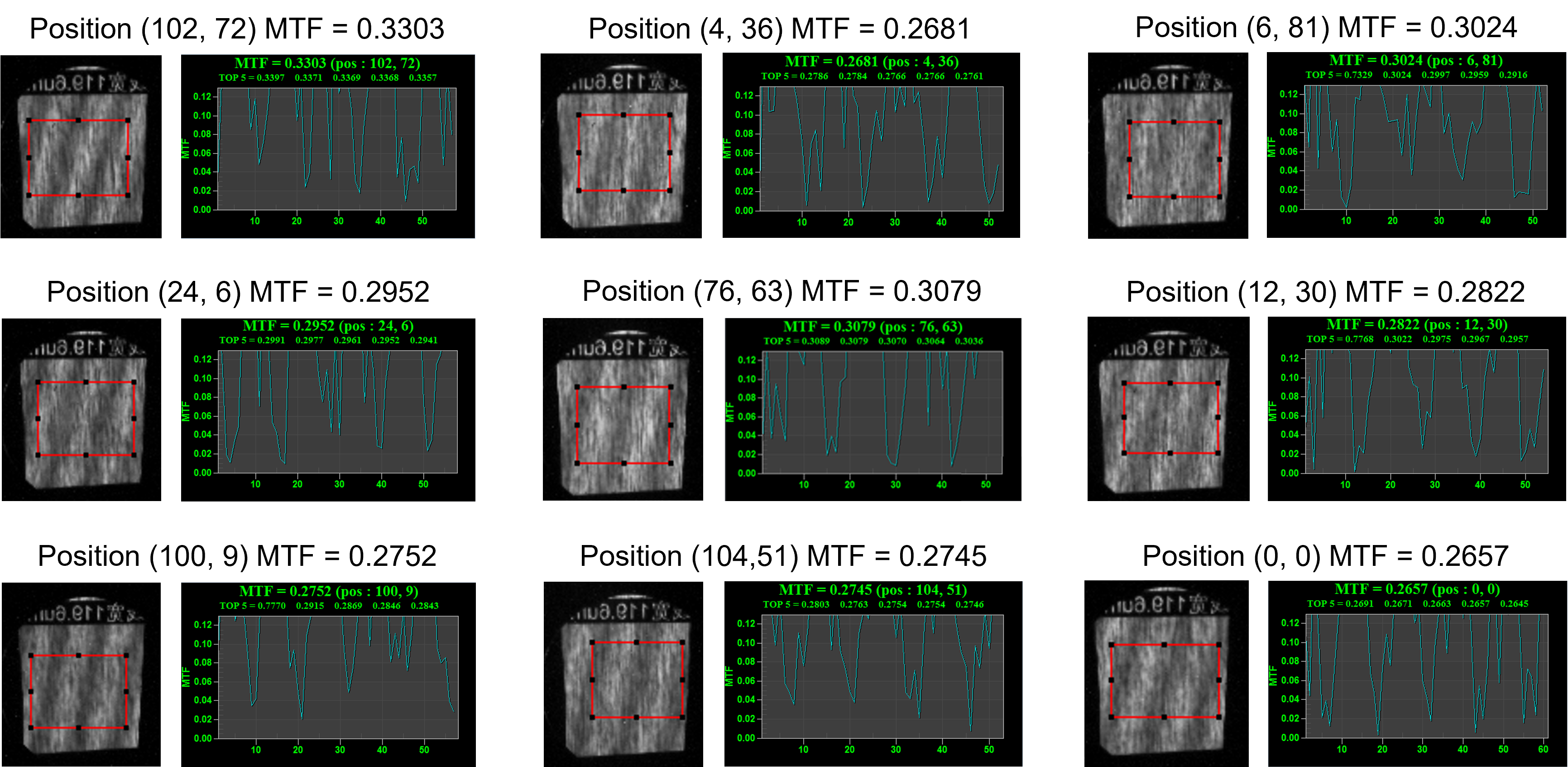


**Fig. S5. MTF tests of the payload on the ground.** Nine regions across the image plane were selected for MTF evaluation (upper-left, upper-center, upper-right, middle-left, center, middle-right, lower-left, lower-center, and lower-right). The left part of each subfigure shows the selected imaging region. The right part shows the MTF variation with the spatial position. The text above each subfigure indicates the MTF at a specified location.

**5. Simulation of “Keystone” and “Smile” effects in snapshot compressive hyperspectral imaging system**

In this section, we present simulation analyses of the “keystone” and “smile” effects in our system. We first introduced “keystone” and “smile” distortions to the original hyperspectral cube, and then encoded the distorted hyperspectral information using a coding matrix, which was subsequently compressed into a two-dimensional measurement. Finally, the generated measurement was reconstructed using the reconstruction network, and the PSNR and SSIM between the reconstructed hyperspectral image and the ground truth were calculated. In this simulation, we only considered the “keystone” and “smile” distortions occurring along the prism dispersion direction (horizontal direction). For simplicity, we define the degree of “keystone” distortion as the amount of pixel shift at the edge position (first row) between 400 nm and 700 nm, denoted as max key. . To define the degree of “smile” distortion, we take 400 nm as the reference wavelength. We then consider the spectral curvature at the edge position (first column), and define the distortion as the pixel offset at the point of maximum curvature, denoted as max smi. . Based on the above definitions, we conducted simulation analyses under three conditions: (1) “keystone” without “smile” (Keystone wo Smile), (2) “smile” without “keystone” (Smile wo Keystone), and (3) both “keystone” and “smile” present (Keystone and Smile). In the simulations, max key. was set to 1, 3, and 5 pixels, and max smi. was also set to 1, 3, and 5 pixels, respectively. The results are shown as Fig. S6.

The figure shows the optical measurement and reconstructed three-band spectral images. From the results, it can be observed that as the “keystone” and “smile” effects increase, the quality of the reconstructed hyperspectral images decreases. Spatial blurring also appears at the edge positions. Therefore, it is significant to take “keystone” and “smile” effects into account in snapshot compressive spectral imaging systems. For


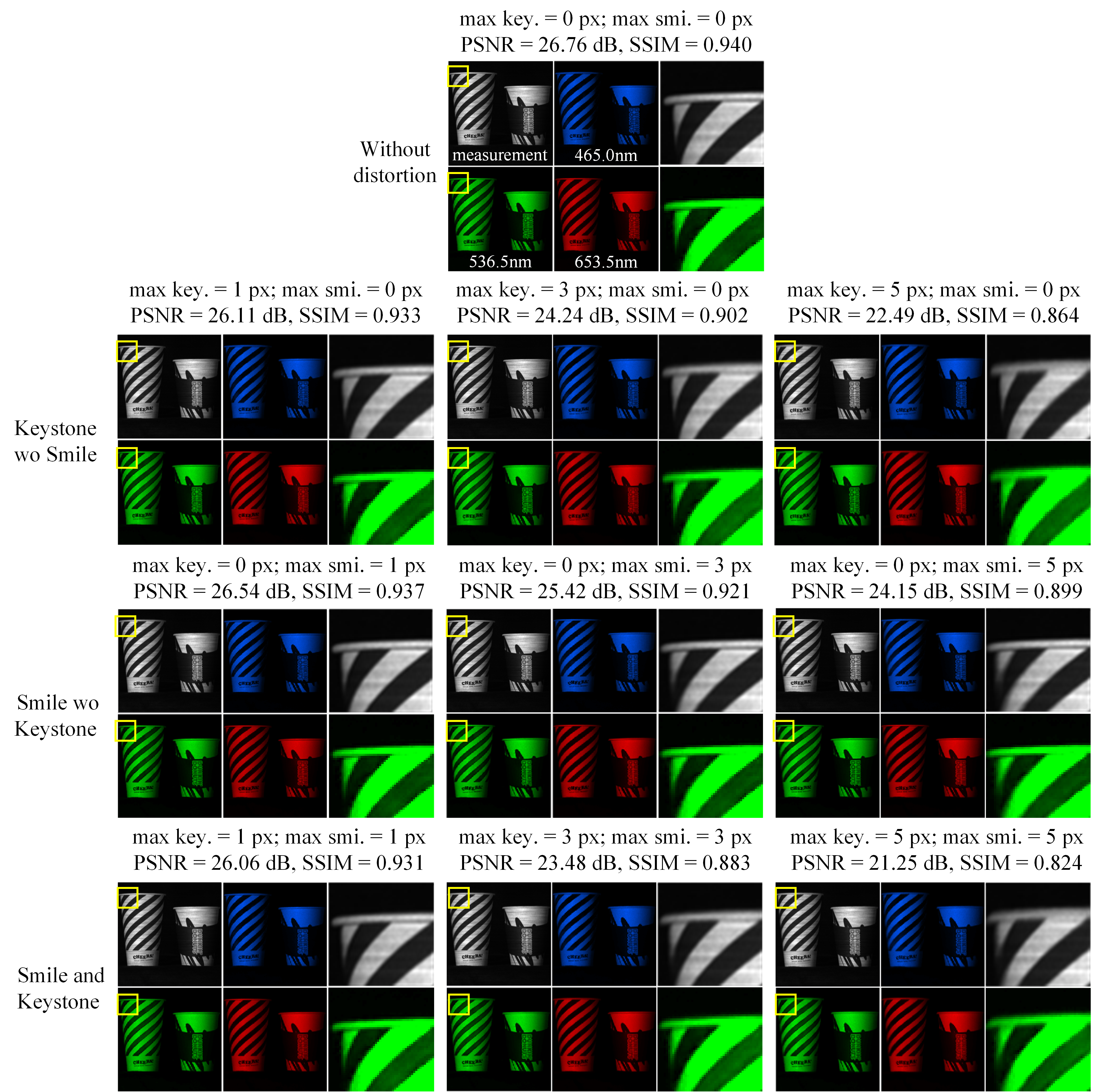


**Fig. S6. Simulation results of “keystone” and “smile” effects**. wo: without. key. : keystone. smi. : smile. px: pixels.

the BUPT-Spectra01 payload proposed in this paper, optical distortions were well corrected during the optical design phase. Although minor “keystone” and “smile” effects may still exist, both ground-based and in-orbit tests demonstrate excellent imaging performance. In future work, we plan to establish a more comprehensive simulation framework for “keystone” and “smile” effects in snapshot compressive hyperspectral imaging systems, to investigate their impact on imaging quality. For the future payloads designs, we will take these two effects into account to optimize both the optical design of the payload and the design of the backend reconstruction algorithms.

**6. Ground test of *BUPT-Spectra01***

Fig. S7a illustrates the transfer function test and focal plane calibration of the payload. First, the focal point of the collimated light tube (1.3 m) was calibrated using a laser interferometer and a flat mirror. Then, an integrating sphere illuminated a stripe target, and the light passed through the collimated light tube into the payload. The stripe target was moved back and forth to find the position where the transfer function was maximized. If this position coincided with the focal point determined in the first step, the focal plane calibration was complete. If there was a discrepancy, the thickness of the focal plane shim was adjusted according to the difference. This process was repeated until the two positions coincided, completing the calibration.

Fig. S7b illustrates the radiometric calibration test. The experiment used a 1-meter diameter integrating sphere. After the integrating sphere was activated, we recorded the imaging results from the sensor under varying parameters (exposure time, gain). Based on the recorded data, correction factors were calculated to compensate for deviations in actual in-orbit imaging.

Fig. S7c illustrates the vacuum test, conducted in a KM6000 vacuum chamber. After the chamber was evacuated to a vacuum state, star-point imaging tests were performed on the payload. In the test, we observed that the star points exhibited a high concentration of energy, which demonstrated the effectiveness of the payload’s vacuum compensation and its operational stability in a vacuum environment.

Fig. S7d illustrates the vibration test of the payload on the DC-6500 vibration platform. We conducted three sets of vibration tests to evaluate the stability of the payload structure. Detailed test parameters are provided in the next section.

**
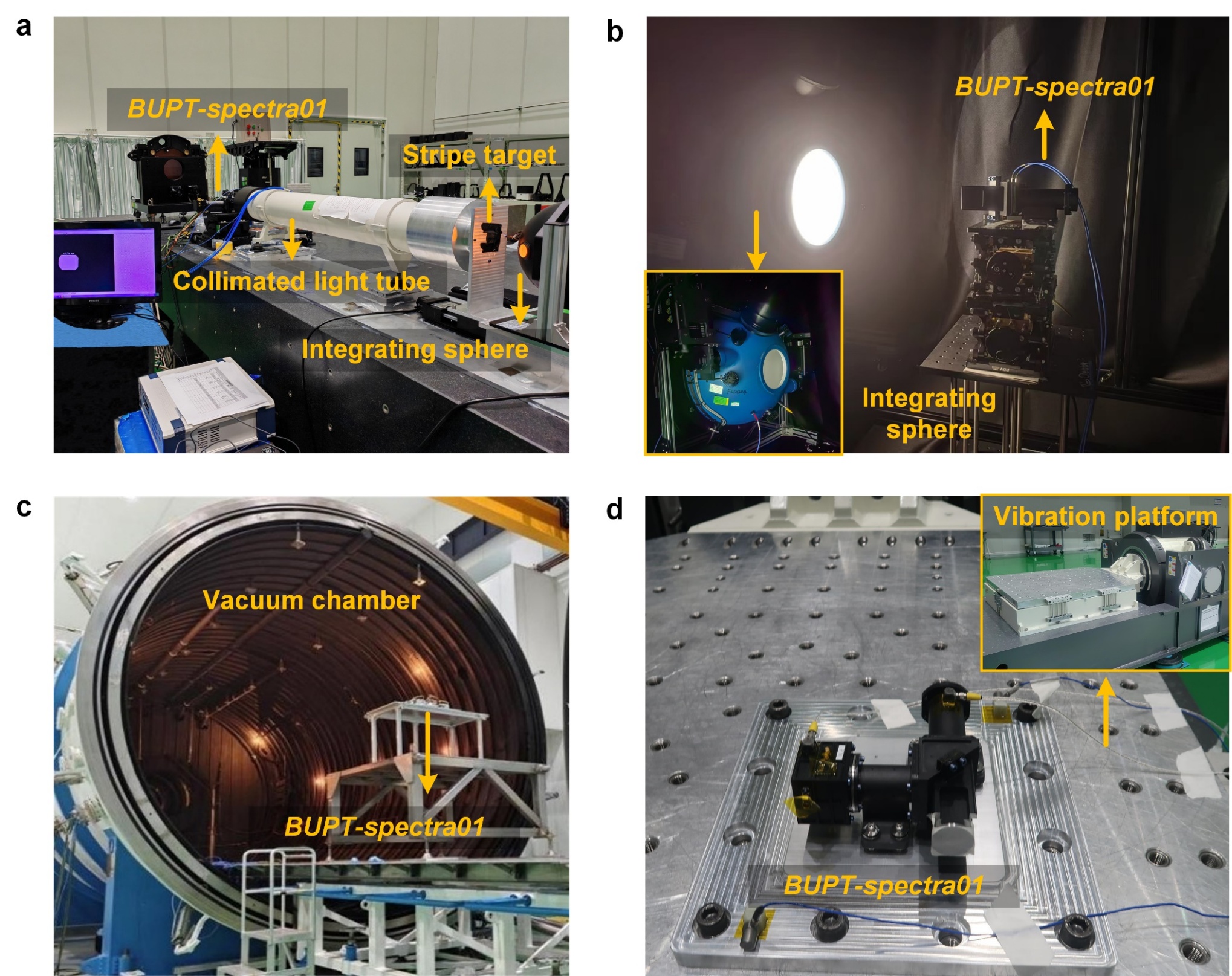
**

**Fig. S7 Ground test. a,** Transfer function test and focal plane calibration. **b,** Radiometric calibration test. **c,** Vacuum test. **d,** Vibration test.

**7. Spatial accuracy of *BUPT-Spectra01***

Fig. S8a shows an outdoor scene captured on the ground. Both the synthesized RGB and spectral images exhibit fine spatial details of the objects. Two regions (I, II) are selected for further spatial evaluation. Fig. S8b displays a vertical grill area, whose orientation is perpendicular to the dispersion direction in the BUPT-spectra01 system. The pixel intensity differences between adjacent grills are significant and clearly distinguishable. Fig. S8c shows a zoomed-in view of the brick area (II), located approximately 100 meters from the system. We can clearly observe the brick contours in this region. These results further demonstrate the excellent spatial performance of the system.


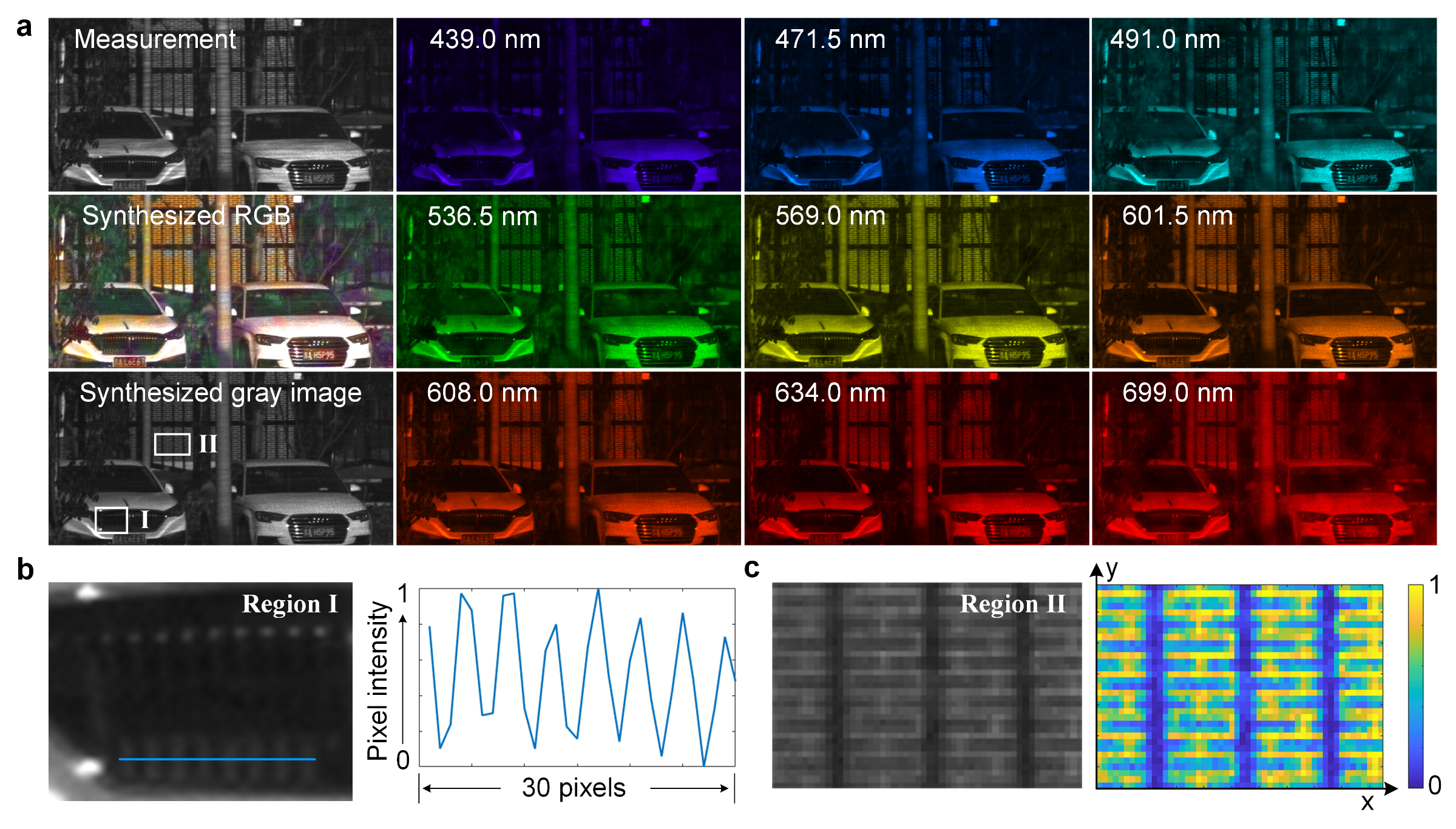


**Fig. S8.** **Spatial accuracy tests of *BUPT-Spectra01*.** **a**, Measurement, synthesized RGB, synthesized grayscale image, and 9-band spectral images of the ground test scene. We select two regions (I, II) for spatial evaluation. **b**, Zoomed-in view of region I. **c**, Zoomed-in view of region II.

**8. Details of the vibration test**

This section presents the parameters for the three sets of vibration tests. Fig. S9a shows the parameter settings for the characteristic sine-sweep vibration test. The vibration platform performed a frequency sweep from 10 Hz to 2000 Hz at a sweep rate of 4 octave $\min^{-1}$ with a vibration amplitude of 0.1 g, where g is the gravitational acceleration. Fig. S9b shows the parameter settings for the sine vibration test. In this test, the vibration platform conducted two tests in each of the X, Y, and Z directions. The first test swept the vibration frequency from 5 Hz to 16 Hz at a rate of 4 octave $\min^{-1}$ with a vibration amplitude of 9.8 mm (o-p), where o-p represents the magnitude from 0 to the peak. The second test swept the vibration frequency from 16 Hz to 100 Hz at a rate of 4 octave $\min^{-1}$. The vibration amplitudes in the X, Y, and Z directions were set to 6 g, 5 g, and 8 g, respectively. Fig. S9c shows the parameter settings for the X/Y/Z three-axis random vibration test. The frequency of the vibration signal changed from 20 Hz to 2000 Hz at a rate of 4 octave $\min^{-1}$. From 20 Hz to 150 Hz, the power of the vibration signal increased by 3 dB for every doubling of the frequency. From 150 Hz to 600 Hz, the power spectral density of the vibration signal was set to 0.04 g² $\mathrm{Hz}^{-1}$. From 600 Hz to 2000 Hz, the power of the vibration signal decreased by 6 dB for every doubling of the frequency. The average intensity of the vibration signal was 6.1 g. During the test, the vibration signal was applied to the X, Y, and Z axes for 120 seconds.


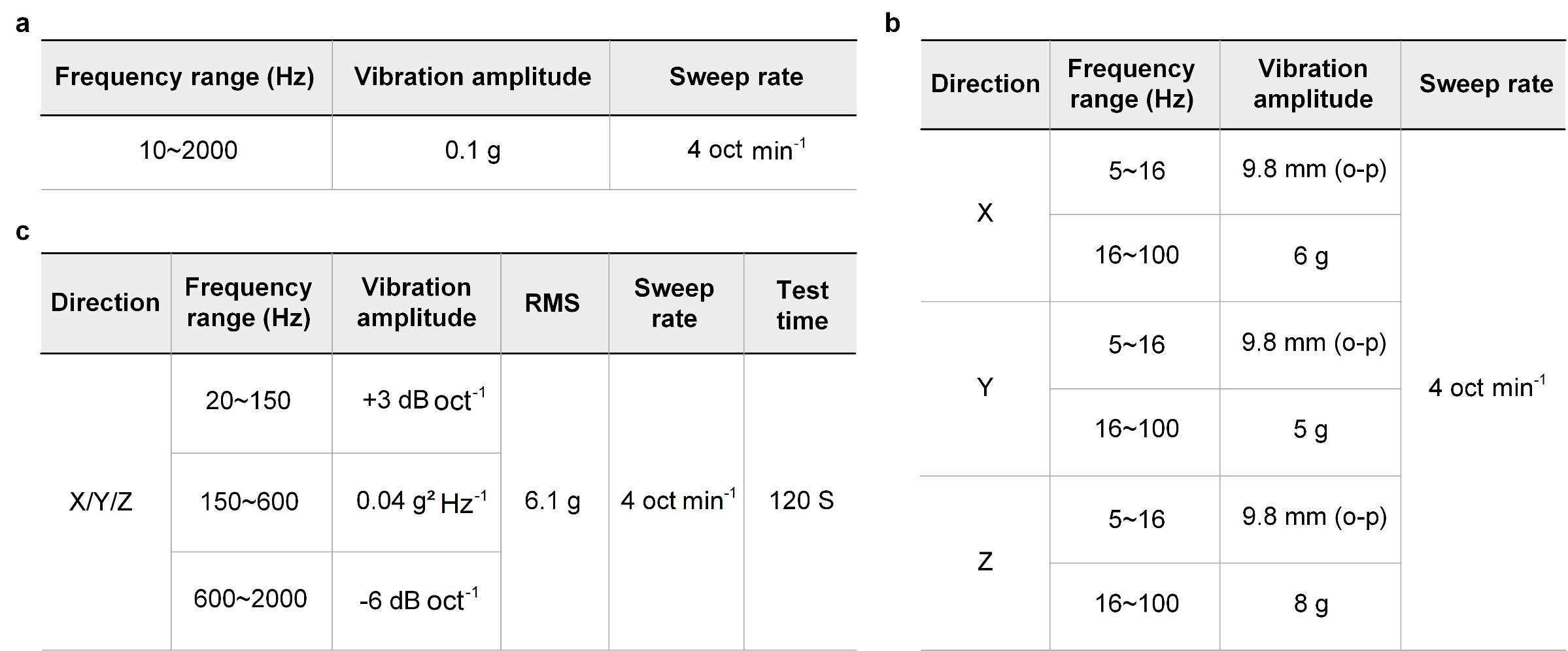


**Fig. S9 Parameter settings for the vibration test. a,** Characteristic sine-sweep vibration test. **b,** Sine vibration test. **c,** X/Y/Z three-axis random vibration test.

**9. Additional in-orbit hyperspectral imaging results**

This section exhibits additional six in-orbit imaging results. In each figure, we show the hyperspectral images (Fig. a), the classification map (Fig. b), the reconstructed spectra (Fig. c), and the PCA analysis (Fig. d).

Fig. S10 shows the hyperspectral imaging results obtained at Kalbarri, Australia. From the classification map, the distribution of forests can be clearly observed, which demonstrate that *BUPT-spectra01* has the potential to be applied for monitoring vegetation coverage. This helps in assessing the status of forest resources and implementing better ecological management. Fig. S11 and Fig. S12 show the hyperspectral imaging results obtained at Teresina, Brazil, and Montes Claros, Brazil, respectively. The ground cover is classified into three categories: forest, city, and cultivated land. We can clearly observe the distribution of cultivated land and its special spectral response. This helps in accurately obtaining the cropland distribution, providing an effective analytical tool for land use planning. Fig. S13 shows the hyperspectral results obtained at a forest in Myanmar. Two kinds of ground cover could be clearly observed: forest and cloud. Analyzing the hyperspectral information of forests helps in assessing the distribution of tree species and promoting forest conservation. Notably, *BUPT-spectra01* enables video-level monitoring of cloud cover, which can be applied in weather analysis. Fig. S14 shows the hyperspectral results obtained at Jinhua, Zhejiang, China. The hyperspectral images and classification results indicate the distribution of buildings, which helps in the analysis of the arrangement of urban population and functional buildings, thereby providing a scientific basis for urban management. Fig. S15 shows the hyperspectral results obtained at a forest in Montevideo, Uruguay. Through spectral analysis, we can clearly observe the natural environment and terrain surrounding the city. This contributes to reasonably planning the direction of urban expansion.


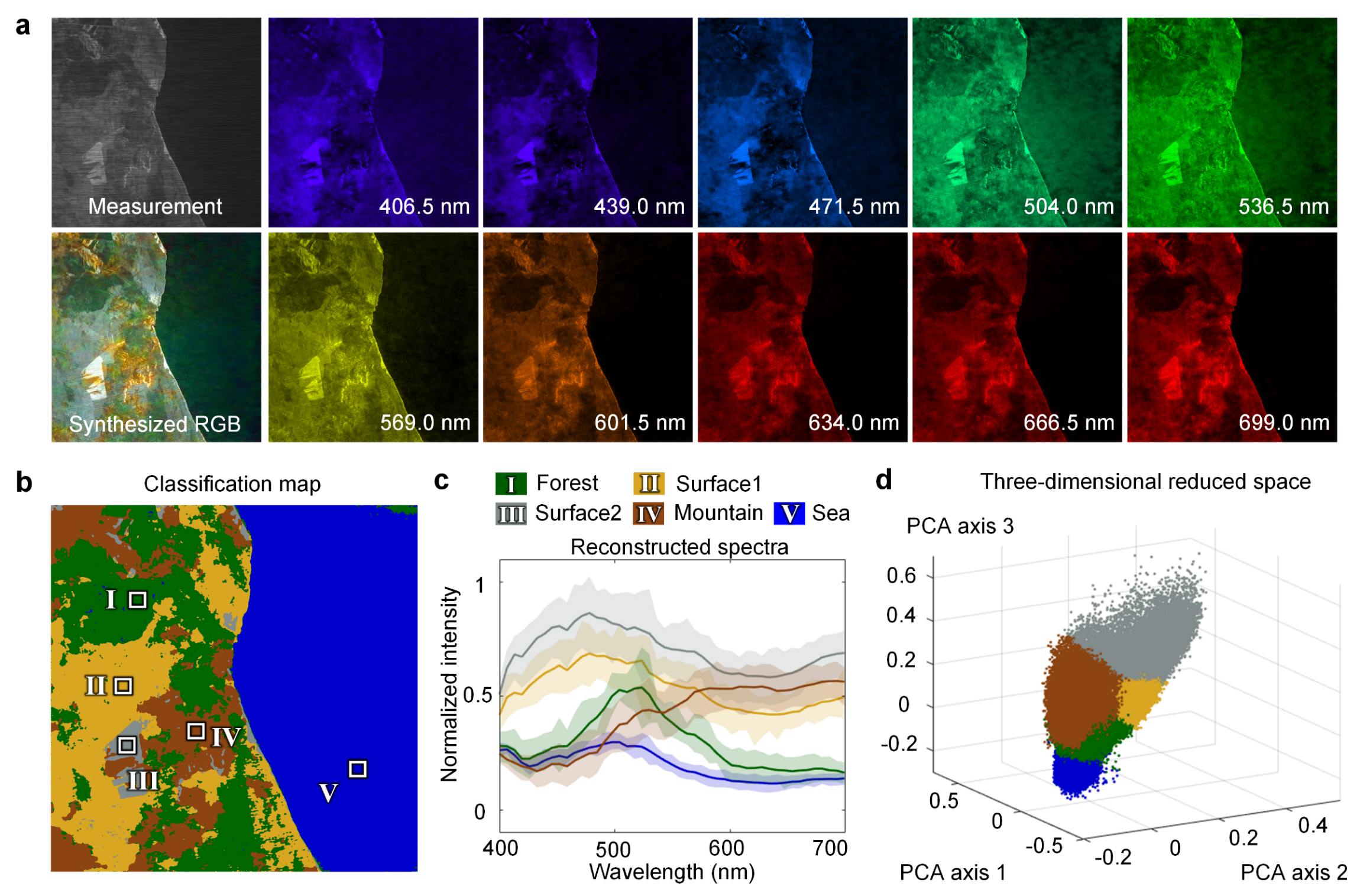


**Fig. S10 In-orbit hyperspectral imaging results of Kalbarri, Australia (27.696° S, 114.210° E). a,** The synthesized RGB image and the spectral images. **b,** The classification map. **c,** The reconstructed spectra. **d,** The PCA analysis.


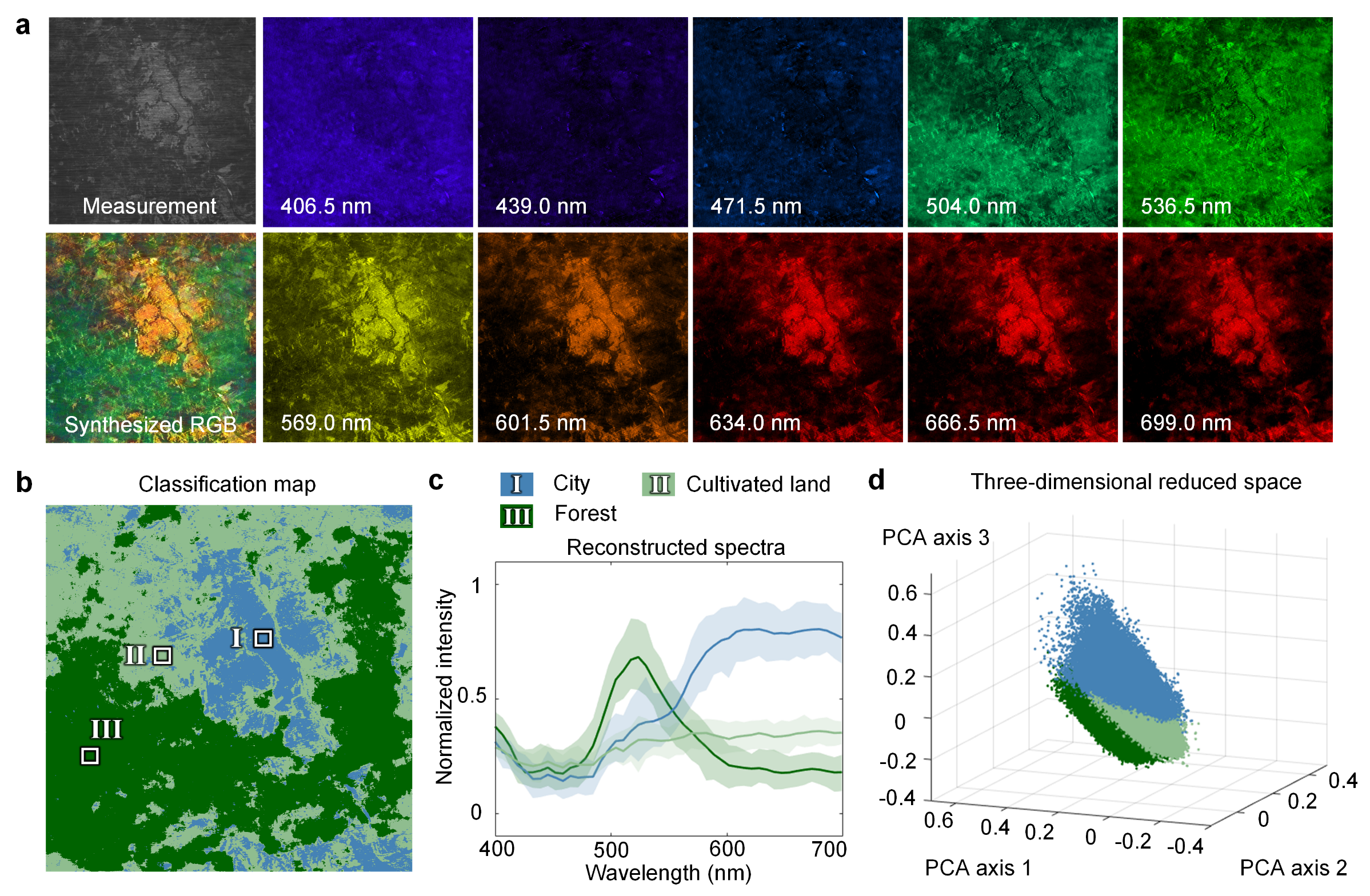


**Fig. S11 In-orbit hyperspectral imaging results of Teresina, Brazil (5.076° S, 42.709° W). a,** The synthesized RGB image and spectral images. **b,** The classification map. **c,** The reconstructed spectra. **d,** The PCA analysis.


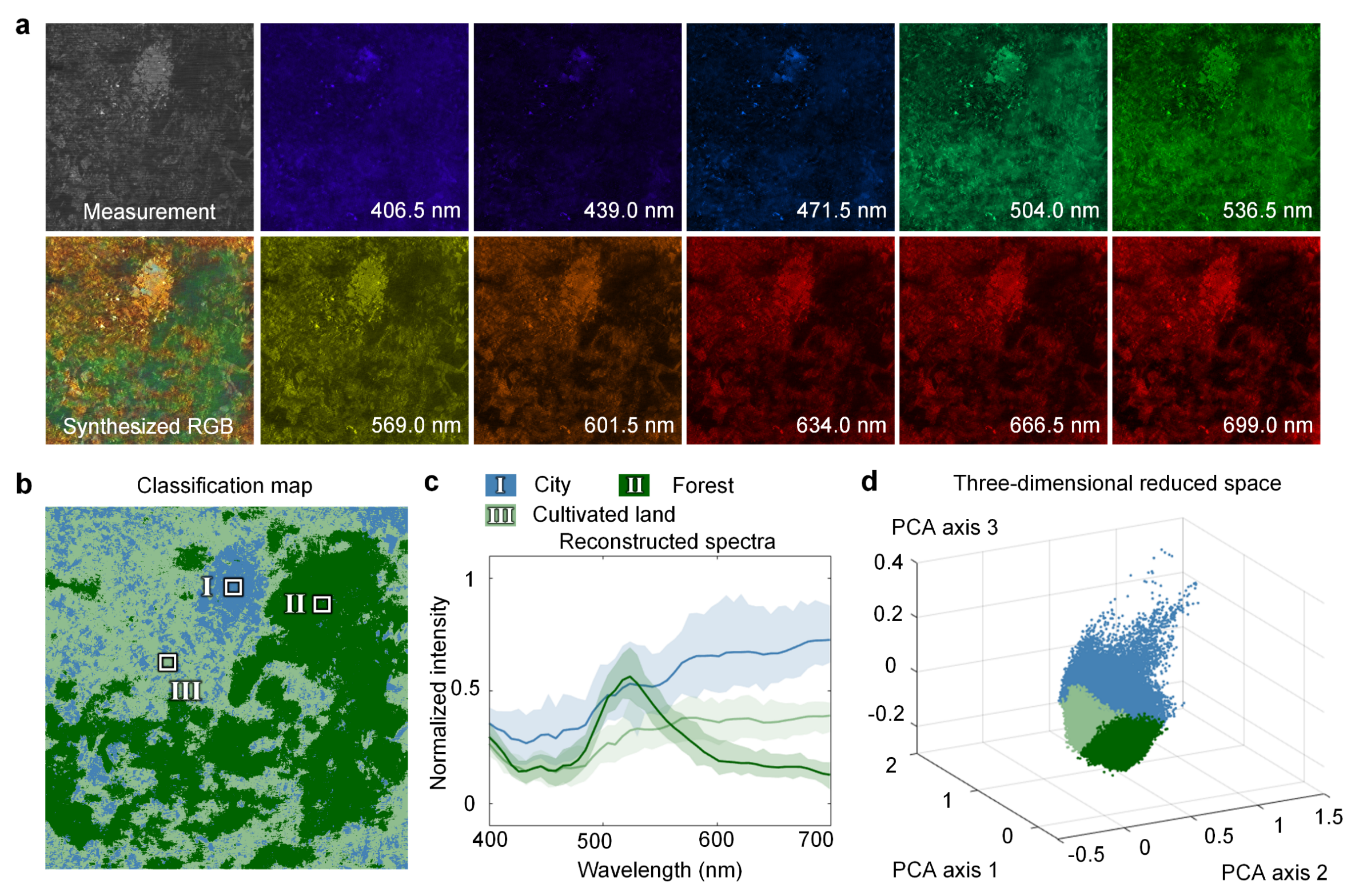


**Fig. S12 In-orbit hyperspectral imaging results of Montes Claros, Brazil (16.324° S, 43.710° W). a,** The synthesized RGB image and spectral images. **b,** The classification map. **c,** The reconstructed spectra. **d,** The PCA analysis.


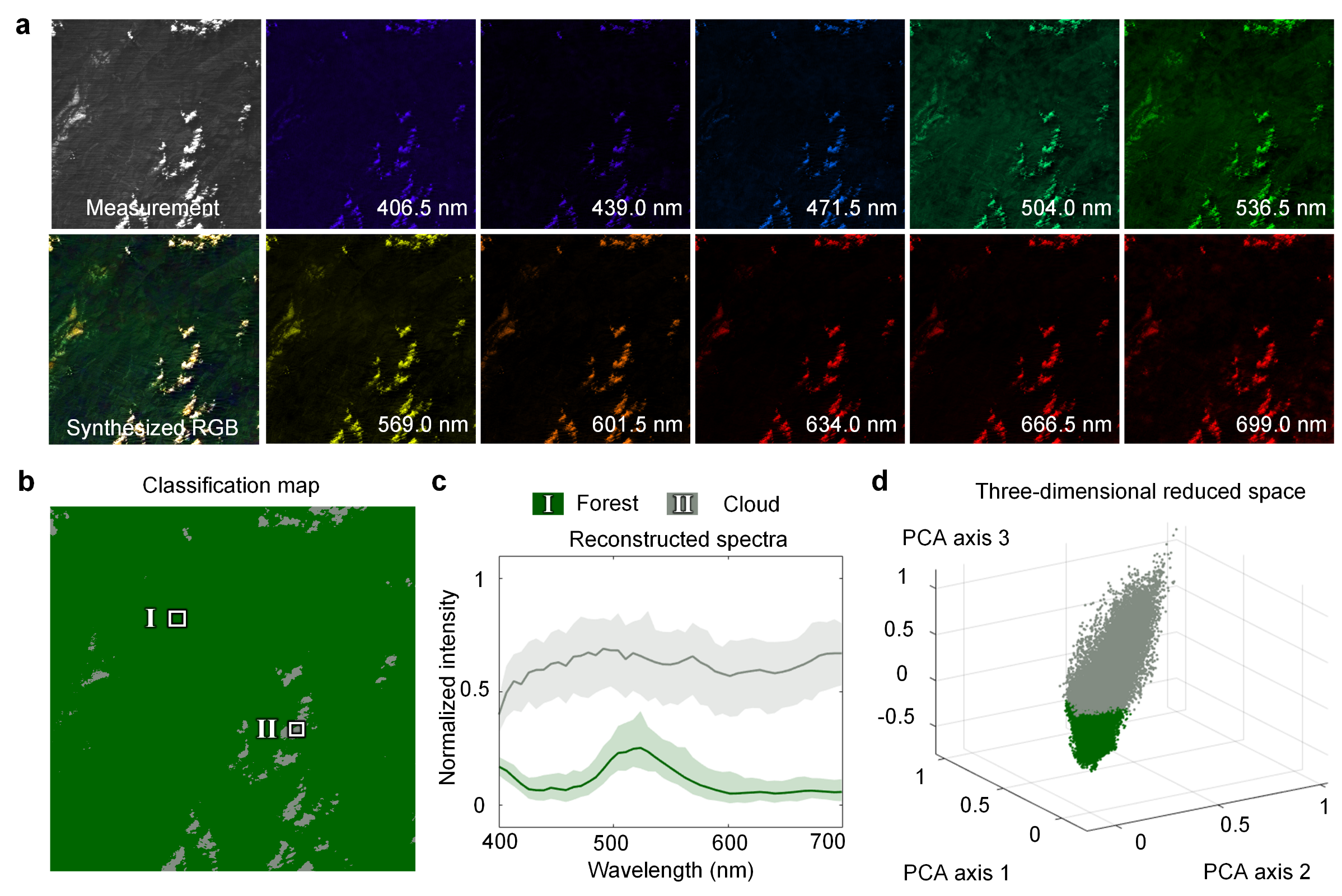


**Fig. S13 In-orbit hyperspectral imaging results of a forest in Myanmar (26.533° N, 95.599° E). a,** The synthesized RGB image and spectral images. **b,** The classification map. **c,** The reconstructed spectra. **d,** The PCA analysis.


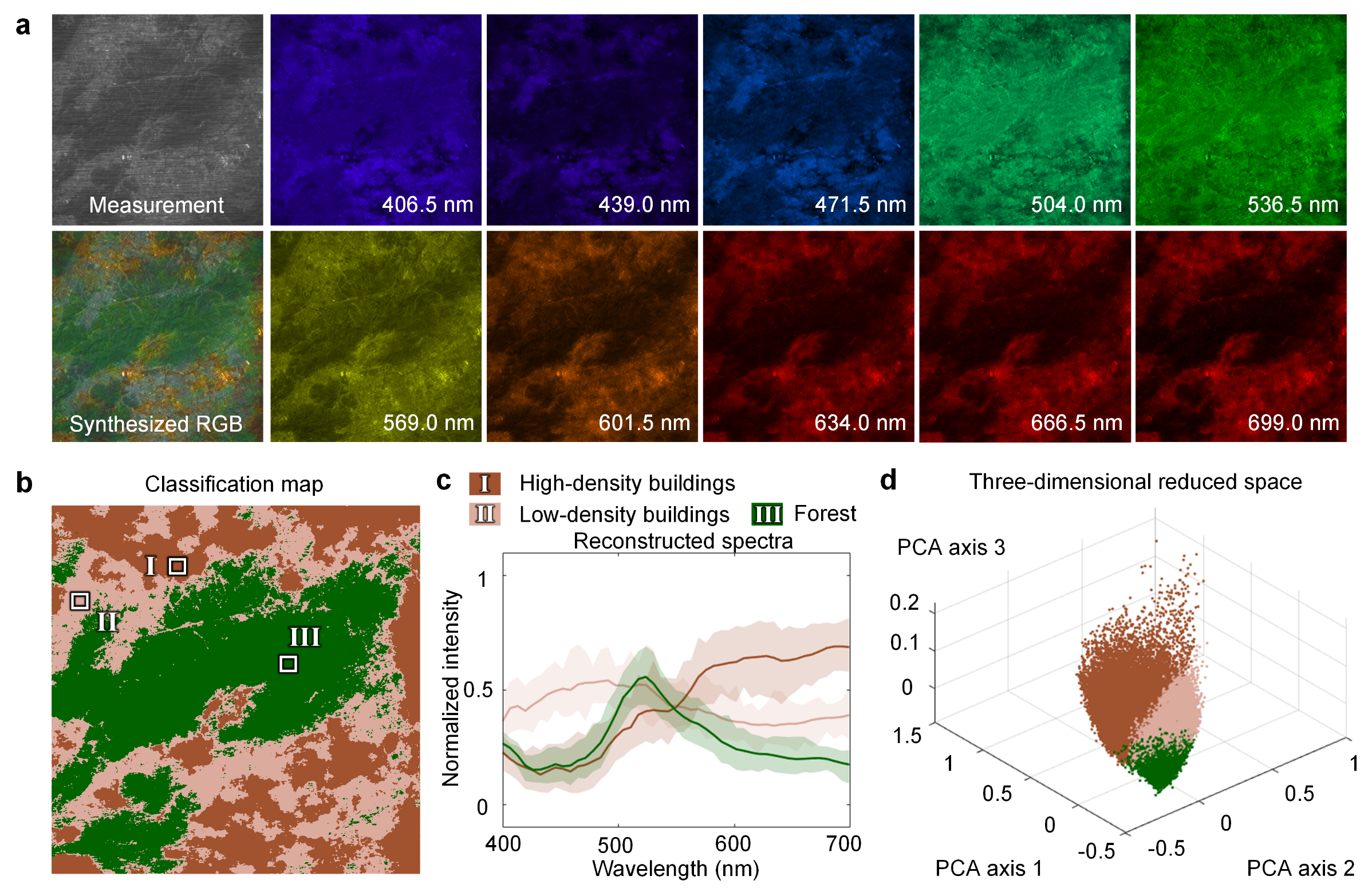


**Fig. S14 In-orbit hyperspectral imaging results of Jinhua, Zhejiang, China (29.080° N, 120.023° E). a,** The synthesized RGB image and spectral images. **b,** The classification map. **c,** The reconstructed spectra. **d,** The PCA analysis.


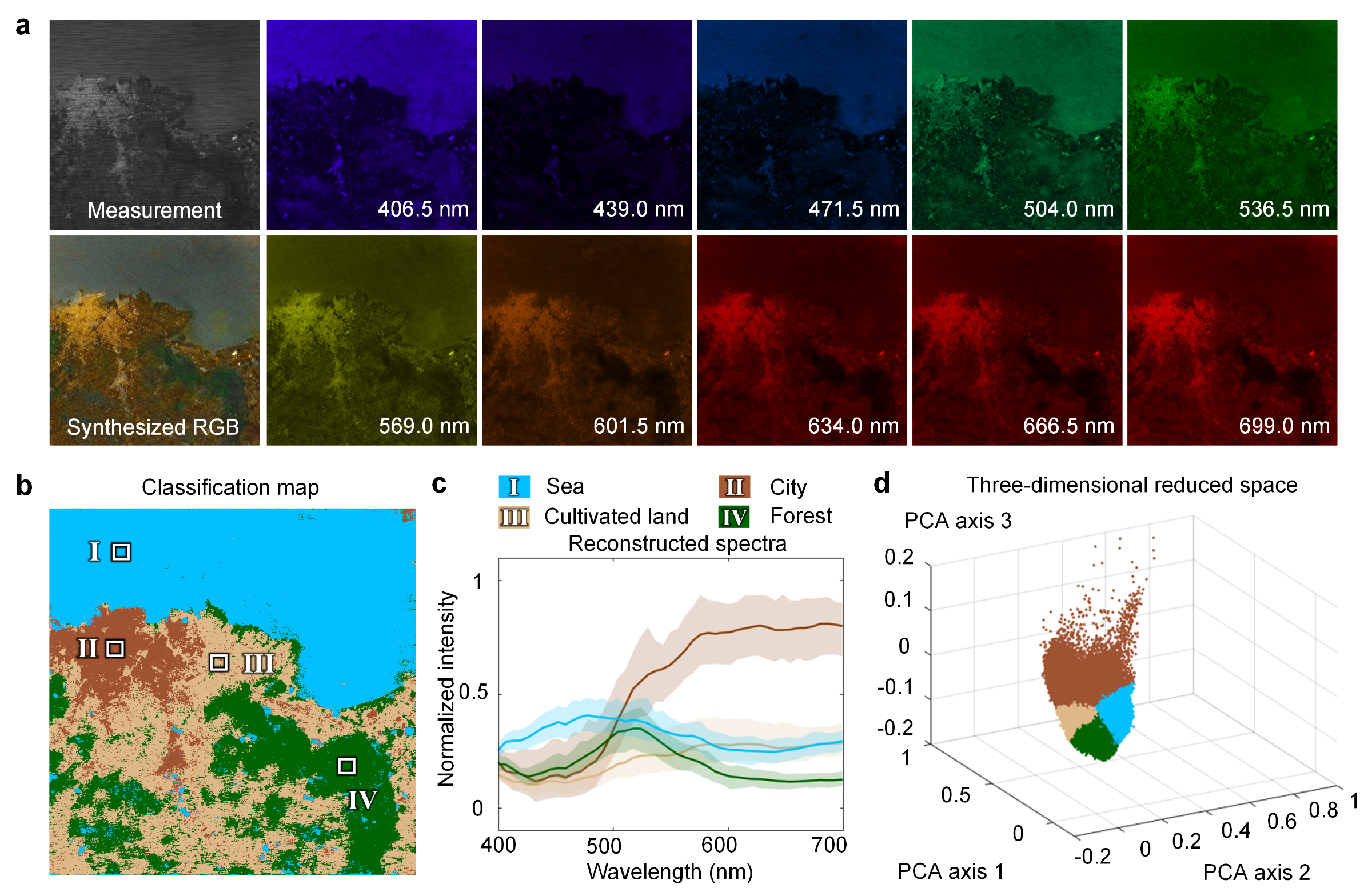


**Fig. S15 In-orbit hyperspectral imaging results of Montevideo, Uruguay (34.606° S, 56.216° W). a,** The synthesized RGB image and spectral images. **b,** The classification map. **c,** The reconstructed spectra. **d,** The PCA analysis.

**10. Classification Accuracy Evaluation**

In this section, we assess the classification accuracy across seven scenarios, as illustrated in Fig. S16. To obtain the ground truth classification maps, we used high-resolution imagery from Google Maps as a reference and invited ten independent annotators to label different ground cover types. For each spatial location, the final class label was determined through majority voting among the annotators. The classification accuracy was then calculated between the predicted classification maps and the human-labeled ground truth. As shown in the results, nearly all scenarios achieve classification accuracies exceeding 80%, demonstrating that BUPT-spectra01 can provide high-quality spatial-spectral images for accurate ground object recognition.


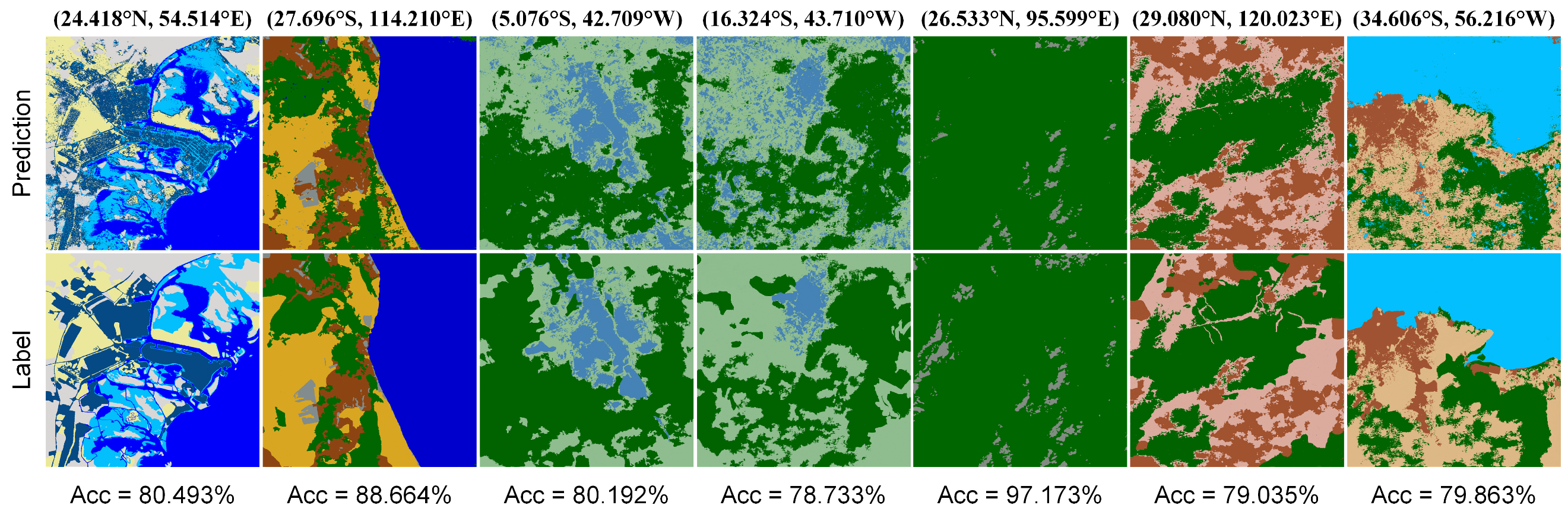


**Fig. S16.** The classification map and human-labeled ground truth of seven scenarios. Acc: Accuracy.

**References**

1. Vaswani, A. et al. Attention is All you Need. *Advances in Neural Information Processing Systems* **30** (2017).
2. Han, K. et al. A Survey on Vision Transformer. *IEEE Transactions on Pattern Analysis and Machine Intelligence* **45**, 87-110 (2022).
3. Park, J. I. et al. Multispectral imaging using multiplexed illumination. IEEE 11th International Conference on Computer Vision, 1–8 (2007).
4. Choi, I. et al. High-quality hyperspectral reconstruction using a spectral prior. In *Technical report*, (2017).
5. Meng, Z. Ma, J. & Yuan, X. End-to-End Low Cost Compressive Spectral Imaging with Spatial-Spectral Self-Attention. 16th European Conference on Computer Vision, 187-204 (2020).
6. Meng, Z. Jalali, S. & Yuan, X. Gap-net for Snapshot Compressive Imaging. *Arxiv preprint*, 2012.08364 (2020).
7. Cai, Y. et al. Mask-Guided Spectral-Wise Transformer for Efficient Hyperspectral Image Reconstruction. IEEE Conference on Computer Vision and Pattern Recognition, 17502-17511 (2022).
8. Hu, X. et al. HDNet: High-Resolution Dual-Domain Learning for Spectral Compressive Imaging. IEEE Conference on Computer Vision and Pattern Recognition, 17542-17551 (2022).
